# Family-Based Haplotype Estimation and Allele Dosage Correction for Polyploids Using Short Sequence Reads

**DOI:** 10.3389/fgene.2019.00335

**Published:** 2019-04-16

**Authors:** Ehsan Motazedi, Chris Maliepaard, Richard Finkers, Richard Visser, Dick de Ridder

**Affiliations:** ^1^Bioinformatics Group, Wageningen University & Research, Wageningen, Netherlands; ^2^Plant Breeding, Wageningen University & Research, Wageningen, Netherlands

**Keywords:** haplotype, polyploid, sequence data, family, estimation

## Abstract

DNA sequence reads contain information about the genomic variants located on a single chromosome. By extracting and extending this information using the overlaps between the reads, the haplotypes of an individual can be obtained. Using parent-offspring relationships in a population can considerably improve the quality of the haplotypes obtained from short reads, as pedigree information can be used to correct for spurious overlaps (due to sequencing errors) and insufficient overlaps (due to short read lengths, low genomic variation and shallow coverage). We developed a novel method, PopPoly, to estimate polyploid haplotypes in an F1-population from short sequence data by taking into consideration the transmission of the haplotypes from the parents to the offspring. In addition, this information is employed to improve genotype dosage estimation and to call missing genotypes in the population. Through simulations, we compare PopPoly to other haplotyping methods and show its better performance. We evaluate PopPoly by applying it to a tetraploid potato cross at nine genomic regions involved in tuber formation.

## 1. Introduction

Genetic polymorphism is the key to understanding inheritance patterns of traits and to identifying genomic regions that affect a trait. While the traits of interest usually have medical importance in human genetics, in plant sciences these traits are often of importance for breeding and selection of the best varieties. Therefore, polymorphic genomic loci are used as genetic markers to investigate co-segregation of genetic variants (alleles) with qualitative traits, e.g., flower color, in populations from crosses or in natural populations. These markers can also be used to investigate the genetic components of quantitative traits such as yield and the degree of tolerance to biotic or abiotic stresses.

The sequence of DNA marker alleles along a single chromosome is called a *haplotype*, of which a diploid organism possesses *k* = 2 versions while a polyploid has *k* > 2. To *phase* markers means to determine these *k* haplotypes, which might be identical (harboring the same alleles) or different (having different alleles at some or all of the marker positions).

Among various types of genetic markers, Single Nucleotide Polymorphism (SNP) markers (Brookes, 1999) are the most abundant and extensively used in genetic studies (Altshuler et al., 2008; Braun et al., 2017). While high-throughput assays such as SNP arrays exist for efficient determination of SNP alleles at single loci, direct determination of haplotypes usually requires laborious and expensive techniques such as bacterial cloning, allele-specific PCR or chromosome microdissection (Michalatos-Beloin et al., 1996; Triplett et al., 2012; Doležel et al., 2014).

However, haplotypes can be used as multi-allelic markers in genetic studies offering more statistical power than single SNPs (Zhang et al., 2002; Simko et al., 2004), as both gene expression and protein function, i.e., the determinants of the phenotypes, can be affected by an allele being in *cis* or *trans* with other alleles (Tewhey et al., 2011). Moreover, a marker allele which is on the same haplotype as a favorable causative allele is likely to be inherited together with that favorable allele, while the co-transmission is unlikely if the alleles are on different haplotypes. This is important for genetic association analysis as well as for marker assisted selection.

Single individual haplotyping (SIH) methods use DNA-sequence reads to phase the SNPs of a single organism at positions covered by the reads, using the fact that the sequence of called alleles should be the same in the reads that originate from the same chromosome. To deal with sequencing errors, which can cause spurious differences between reads of the same chromosome and therefore can influence variant calling and haplotyping especially at low sequencing depths, these methods use probabilistic models or cost functions to prefer a certain phasing to others based on the observed reads (Bansal and Bafna, 2008; Aguiar and Istrail, 2013; Berger et al., 2014; Das and Vikalo, 2015; Lancia, 2016; Xie et al., 2016).

Recently, algorithms have been proposed that apply the rules of Mendelian inheritance to combine the information of reads and transmission in a cross in a cost function for diploids (Garg et al., 2016) or in a probabilistic model with arbitrary ploidy levels (Motazedi et al., 2018a). However, both of these approaches focus on trios consisting of two parents and one offspring, and therefore ignore the information provided by larger populations. In cross populations, the number of haplotypes is usually limited by the set of parental haplotypes, and therefore it is expected that we detect multiple occurrences of each haplotype across the population. This a priori information can be used to ease the estimation of haplotypes (Stephens et al., 2001), but is not taken into account by the current methods. In addition, these methods accept recombinant haplotypes in the phasing estimate of the offspring (with the recombination cost/probability being preset as desired), while recombination events have a very low probability between loci that are only a few thousands nucleotides apart, i.e., in the typical range of haplotypes obtained from short sequence reads. Sequencing and genotype calling errors can therefore be misinterpreted as recombination events by these methods and thus result in spurious haplotypes, especially in polyploids.

Here we propose a new haplotype estimation algorithm, PopPoly, that specifically targets larger F1-populations, which consist of two parents and several offspring, sequenced by short read sequencing technologies. Considering the short length of the reads, and hence the limitation of read-based phasing to a few hundreds to thousands of nucleotides, PopPoly is based on the assumption that all of the population haplotypes must be present in the parents. Therefore, all of the population reads are combined to estimate the parental haplotypes using a Bayesian probabilistic framework in the first step, and the offspring haplotypes are selected from the estimated parental haplotypes using the minimum error correction (MEC) criterion (Lippert et al., 2002). In addition, PopPoly uses the inheritance information to detect and correct wrongly estimated SNP dosages and to estimate missing genotypes in the population.

Through simulations of potato crosses with varying numbers of offspring and sequencing depths, we compare PopPoly to other haplotype estimation methods and show that it improves phasing and variant calling accuracy. Furthermore, two parents and 10 offspring of a potato cross were sequenced and subsequently analyzed by PopPoly for 9 loci. For one of these loci (*StFKF1*), we selected haplotype tagging SNPs (*ht*SNPs) for the eight haplotypes proposed by PopPoly and developed a KASP assay (Semagn et al., 2014) to assess the segregation in an offspring population of 181 individuals. Using genetic rules, we validated the haplotype solution proposed by PopPoly.

## 2. Materials and Methods

Short-read sequencing technologies, such as Illumina, produce high-quality sequence reads of up to a few hundred bases in length, which are randomly positioned over the target genomic region and together cover each target position multiple times. By aligning these reads to some consensus reference, genomic variations can be detected and the variant alleles can be specified within each read. To resolve the succession of genomic variants on each chromosome, haplotype estimation or haplotyping methods aim to group the reads that have the same variants at the same positions as originating from the same chromosome. This approach requires overlap of the reads at the variation sites and the inclusion of at least *two* variation sites in a read, so that the flanking positions can be connected by the overlaps in between.

However, some of the reads do not meet the criterion of containing at least two variation sites, and the connection between the variation sites can be therefore broken at some positions. For this reason, current haplotyping algorithms start by detecting positions connected to each other through the sequence reads and aim to resolve the haplotypes over each obtained set of connected positions, i.e., the so-called “haplotype blocks” or solvable islands. With short sequence reads, haplotype blocks often include a few hundred up to a few thousand bases.

In our approach, we use the fact that recombination events are usually extremely unlikely over the short distances covered by the haplotype blocks obtained from short reads. This usually confines the haplotypes observed in an F1 generation of small to moderate size to the haplotypes that exist in its parental cross. Assuming each parent transmits half of its chromosomes at random to its progeny, we combine all of the reads in an outcrossing F1-population that consists of two heterozygous parents and their F1 offspring, to estimate the haplotypes of the parents and determine the haplotypes of each offspring by selecting the phasing most compatible with its reads from the set of phasings offered by the transmission of the (already estimated) parental haplotypes.

**Figure 1:**
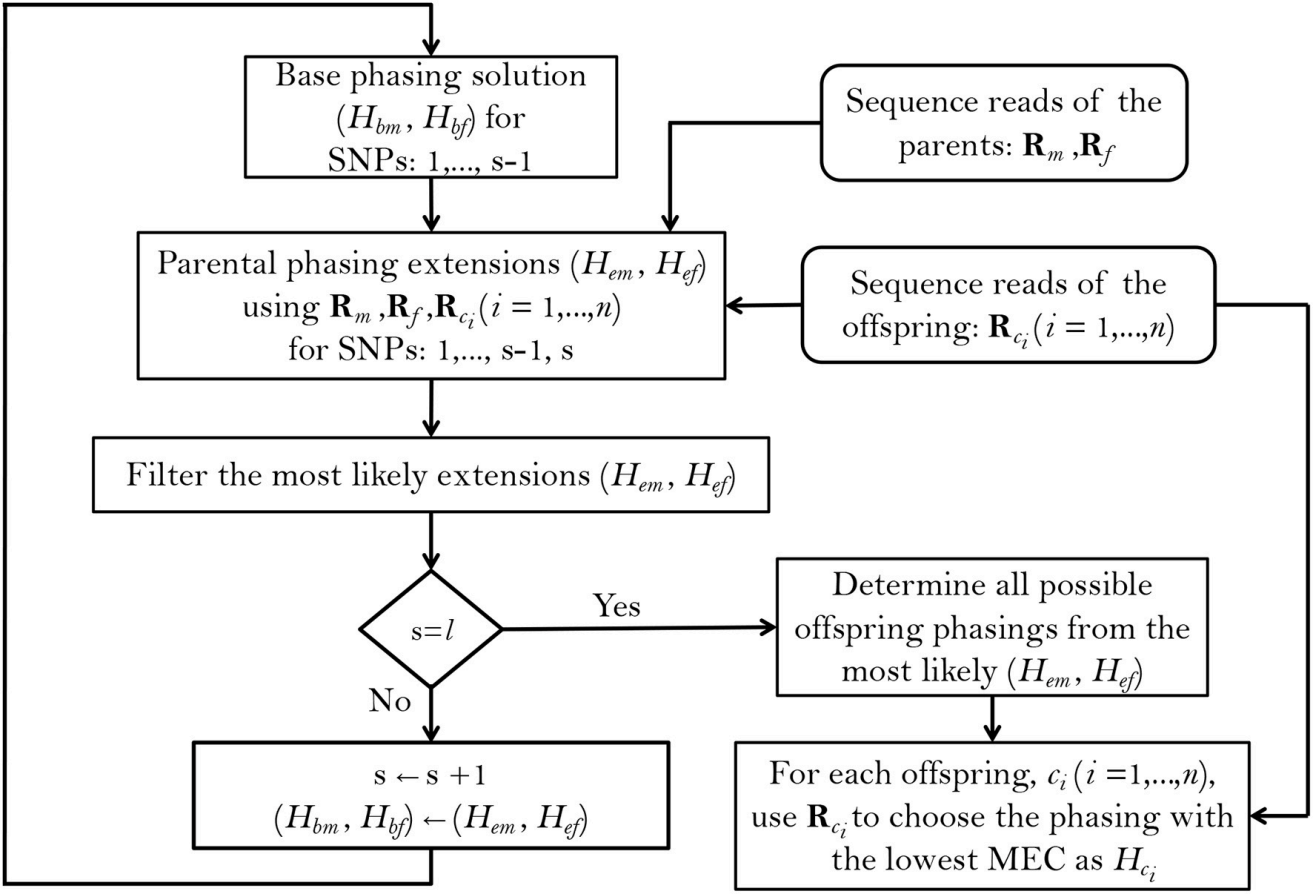
Summary of the PopPoly method to estimate haplotypes in an F1-population with two parents, (*m, f*), and *n* offspring, *c*_*i*_ (*i* = 1, …, *n*), using the sequence reads for a block including *l* SNPs.

To implement this method, we follow a greedy SNP-by-SNP extension approach (Figure 1), extending the base phasings *H*_*bm*_ and *H*_*bf*_ (for the mother and father, respectively) at each step by one SNP and choosing the most likely phasing extensions *H*_*em*_ and *H*_*ef*_ to continue with, as the base phasings of the next step, until all of the *l* SNPs within a haplotype block have been phased. Starting by the first two SNP positions in the block, the probabilities of the base and extended parental phasings, conditional on the reads and taking the observed offspring genotypes into account, are calculated using Bayes' formula. We use *s* = 2 to *s* = *l* to denote the current extension SNP (as the starting base phasing is just the SNP genotype at *s* = 1), and denote the phasing extensions and called SNP genotypes by $H_{m}^{s}$, $H_{f}^{s}$, $H_{c_{i}}^{s}$ and $G_{m}^{s}$, $G_{f}^{s}$, $G_{c_{i}}^{s}$ for mother, father and offspring *c*_*i*_ (*i* = 1, ⋯ , *n*) respectively. With these notations, the probability of each possible parental extension at *s* is related to its base phasing at *s*−1 according to:

(1) $$\begin{array}{l} P(H_{m}^{s},H_{f}^{s}|H_{m}^{s-1},H_{f}^{s-1},G_{m}^{s},G_{f}^{s},G_{c_{1}}^{s},\ldots ,G_{c_{n}}^{s},R_{set},\epsilon_{set})=\\ \frac{P(R_{set}|H_{m}^{s},H_{f}^{s},\epsilon_{set})P(H_{m}^{s},H_{f}^{s}|G_{m}^{s},G_{f}^{s},G_{c_{1}}^{s},\ldots ,G_{c_{n}}^{s},H_{m}^{s-1},H_{f}^{s-1})}{\sum_{\left(H_{m}^{s},H_{f}^{s}\right)\prime }P\left(R_{set}|\left(H_{m}^{s},H_{f}^{s}\right)\prime ,\epsilon_{set}\right)P\left(\left(H_{m}^{s},H_{f}^{s}\right)\prime |G_{m}^{s},G_{f}^{s},G_{c_{1}}^{s},\ldots ,G_{c_{n}}^{s},H_{m}^{s-1},H_{f}^{s-1}\right)} \end{array}$$

where **R**_*set*_ denotes the set of all of the reads in the population and **ϵ**_*set*_ stands for the set of base-calling error vectors, ϵ_*j*_, associated with each *r*_*j*_ ∈ **R**_*set*_ (1 ⩽ *j* ⩽ |**R**_*set*_|). $P\left({\text{R}}_{set}|H_{m}^{s},H_{f}^{s},\epsilon_{set}\right)$ denotes the conditional probability of observing the reads given a pair of maternal and paternal extensions at *s*, $\left(H_{m}^{s},H_{f}^{s}\right)$, and the base-calling error probabilities given by **ϵ**_*set*_.

The details of calculating Equation 1 are given in Appendix A (Supplementary Material). In order to get rid of improbable extensions and keep the number of stored phasings (almost) constant at each stage of the algorithm, at each *s* we discard those extensions that have a posterior probability less than 0 < ρ ≤ 1, i.e., we apply *branching* with hard thresholding. We then *prune* further the remaining extensions using a soft threshold 0 ≤ κ ≤ 1 by discarding those with a posterior probability less than κ*P*_*max*_, where *P*_*max*_ denotes the maximum posterior probability among the branched extensions (Berger et al., 2014; Motazedi et al., 2018a). The values of ρ and κ can be given by the user, and were set to 0.2 and 0.94, respectively, in our simulations.

This Bayesian framework for phasing extension can also be used to detect erroneous SNP genotypes, which result in zero probabilities for all extensions at a SNP position. We use a similar Bayesian approach to re-estimate these erroneous genotypes, as well as the uncalled SNP genotypes of the parents, by assigning probabilities to the possible population genotypes at a SNP position conditional on the reads and the segregation of parental alleles at the SNP position according to:

(2) $$\begin{array}{l} P(G_{m}^{s},G_{f}^{s},G_{c_{1}}^{s},\cdots ,G_{c_{n}}^{s}|R_{set},\epsilon_{set})=P(G_{c_{1}}^{s},\cdots ,G_{c_{n}}^{s}|G_{m}^{s},G_{f}^{s},R_{set},\epsilon_{set})\\ \;P(G_{m}^{s},G_{f}^{s}|R_{set},\epsilon_{set}) \end{array}$$

In order to calculate Equation (2), we first obtain the posterior probabilities of the parental genotypes, $P\left(G_{m}^{s},G_{f}^{s}|{\text{R}}_{set},\epsilon_{set}\right)$, in a manner similar to that used in obtaining extension probabilities (Equation 1). We then assume conditional independence of the offspring genotypes given the parents, i.e., their exchangeability, to calculate:

(3) $$\begin{array}{l} P(G_{c_{1}}^{s},\cdots ,G_{c_{n}}^{s}|G_{m}^{s},G_{f}^{s},{\text{R}}_{set},\epsilon_{set})=P(G_{c_{1}}|G_{m}^{s},G_{f}^{s},{\text{R}}_{c_{1}},\epsilon_{c_{1}})\cdot \;\ldots \;\cdot \\ \;P(G_{c_{n}}|G_{m}^{s},G_{f}^{s},{\text{R}}_{c_{n}},\epsilon_{c_{n}}) \end{array}$$

The details of calculating Equations (2) and (3) are given in Appendix B (Supplementary Material). The set of population genotypes with the highest likelihood is then assigned to each individual and used in Equation (1) for phasing extension.

After obtaining surviving phasing extensions at the last SNP position *s* = *l*, a phasing is chosen for each offspring from each set of parental phasing estimates by looking into the possible transmissions of the parental *l* SNP haplotypes. Assuming each parent transmits half of its haplotypes to each offspring, which of course requires balanced meiosis and even ploidy levels, $\left(\begin{array}{c} \frac{k_{m}}{2}\\ 2 \end{array}\right)\cdot \left(\begin{array}{c} \frac{k_{f}}{2}\\ 2 \end{array}\right)$ offspring phasings will be possible from each set of parental estimates, with *k*_*m*_ and *k*_*f*_ being the ploidy levels of the mother and the father, respectively. From this set of candidate phasings, we assign to each offspring the phasing that yields the smallest minimum error correction (MEC) score with respect to its individual sequence reads (Lippert et al., 2002) (Appendix C in Supplementary Material).

Finally, each set of parental estimates and the offspring phasings deduced from them is ranked according to the relative likelihood of the parental phasings (compared to the other surviving phasings of the parents) and the sum of the MEC scores of the deduced offspring phasings. Thus, the output of the algorithm consists of sets of ranked phasing estimates for the whole population. In our simulations, we only kept the best set of population estimates for evaluation and comparison with other methods.

To examine the computational complexity of PopPoly and to see how it scales with respect to the maximum sequencing depth $d_{max}=\max\left(d_{m},d_{f},\overset{n}{\underset{i=1}{\max}}d_{c_{i}}\right)$ (with *d*_*m*_, *d*_*f*_ and *d*_*c*_*i*__ representing the sequencing depths of the mother, father and offspring *c*_*i*_, respectively), population size *n* + 2, and the number of SNPs *l* in the region of interest, we assume that the number of surviving extensions is effectively constant at each stage of the algorithm and denote it by η. Setting *k* = max(*k*_*m*_, *k*_*f*_), for each of the η base phasings at most (*k*!)^2^ extensions must be examined at each extension step. For each of these extensions, Equation (1) requires $O\left(\left(n+2\right)d_{\max}\right)$ calculations. To call the genotypes at a SNP position, Equation (2) requires calculations of the order $O\left({\left(k+1\right)}^{2}d_{max}^{2}n\right)$, as the dosage of the alternative allele can vary from 0 to *k* in each parent (resulting in $O\left({\left(k+1\right)}^{2}d_{max}\right)$ complexity for the number of possible parental genotypes) and for each candidate pair of parental genotypes $O\left(d_{max}\right)$ calculations are needed in each offspring to obtain the likelihood of its genotype conditional on the sequencing reads and the pair of parental genotypes (Equation 3). This adds up to:

(4) $$\begin{array}{l} O\left(\eta {\left(k!\right)}^{2}(n+2)d_{max}^{2}\right) \end{array}$$

complexity at each extension step. Multiplying the explained complexity by *l*, i.e., the number of extension steps, leads to the computational complexity of estimating parental phasings. The selection of offspring phasings using MEC scores at the end requires $O\left(n{\left(\begin{array}{c} \frac{k}{2}\\ 2 \end{array}\right)}^{2}ld_{max}\right)$ calculations for each surviving pair of parental estimates. Using (k2)2<k! and *n* + 2 ≤ 3*n* (as *n* ≥ 1), the total complexity is:

(5) $$\begin{array}{l} O\left(n\eta l{\left(k!\right)}^{2}d_{max}^{2}\right) \end{array}$$

which increases linearly with the number of SNPs *l* and the number of offspring *n* and quadratically with the sequencing depth *d*_*max*_.

### 2.1. Performance Evaluation by Simulation

To evaluate the performance of PopPoly and compare it to other haplotyping methods, we simulated genomic regions for bi-parental F1-populations of tetraploid potato, as described in Motazedi et al. (2018a). We simulated different scenarios, varying the number of offspring from 1 to 30. For each scenario, we randomly selected 100 regions of length 1 *kb* from the chromosome 5 sequence of the PGSC potato reference genome (release version 4.04) (Potato Genome Sequencing Consortium, 2011). The genomes of the two parents were independently obtained for each region by introducing on average one bi-allelic SNP per 50 bp (*SD* = 90 bp) according to the lognormal SNP density model and the dosage distributions described in Motazedi et al. (2018b), determined using data from a panel of tetraploid potato cultivars (Uitdewilligen et al., 2013). To simulate each offspring, two chromosomes were randomly selected from each parent. For the potato genome, typical ratios of genetic to physical distance vary in the range of 3 to 8 *cM*/*Mb* in different regions (Felcher et al., 2012; Bourke et al., 2015). Therefore, the assumption of improbable recombination holds for the simulated genomic regions and population sizes.

For each simulated population, paired-end Illumina HiSeq 2000 reads were generated *in silico*, with an average insert-size of 350 bp and single read length of 125 bp, using the sequencing simulator ART (Huang et al., 2011). The simulated sequencing depth was 5× per homolog for each parent and 2× per homolog for the offspring. We also conducted simulations of families with 2, 6 and 10 offspring with higher sequencing depths, up to 30× per homolog for each individual, in order to evaluate the performance at higher coverages.

After mapping the simulated reads to their reference regions using BWA-MEM (Li, 2013) and calling SNPs using FreeBayes (Garrison and Marth, 2012), we estimated the phasing of the parents and the offspring in each F1-population using state-of-the-art SIH methods: SDhaP (Das and Vikalo, 2015) and H-PoP (Xie et al., 2016), for comparison to PopPoly. We chose these two methods because of their computational efficiency and their allowing for SNP dosage correction, as well as the shown higher accuracy of H-PoP compared to the other state-of-the-art SIH methods (Xie et al., 2016). We also estimated the haplotypes using the trio based method available for polyploids: TriPoly (Motazedi et al., 2018a), and compared the obtained estimates to those obtained by PopPoly and the SIH methods.

We used several measures to compare the accuracy of haplotype estimation with the used methods. These include the *pair-wise phasing accuracy rate* (*PAR*), defined as the proportion of correctly estimated phasings for SNP-pairs (Motazedi et al., 2018b), as well as the *reconstruction rate* (*RR*) defined to measure the overall similarity between the original haplotypes and their estimates using the Hamming distance (Motazedi et al., 2018a).

As the quality of haplotype estimation depends not only on the accuracy of the estimated haplotypes, but also on the ability of the haplotyping method to phase as many SNPs as possible and to efficiently handle missing SNPs and wrong dosages, we calculated the *SNP missing rate* (*SMR*) and *incorrect dosage rate* (*IDR*) in the estimated haplotypes for each method.

Finally, to evaluate the continuity of phasing we measured the average number of phasing interruptions, i.e., the number of haplotype blocks minus one, in the estimates of each method and normalized it by the number of SNPs, *l*, as *number of gaps per SNP* (*NGPS*). The number of haplotype blocks for a set of SNPs, S, is equal to the number of connected components in the *SNP-connectivity graph*, $G_{S}=(S,E_{S})$, in which each node represents a SNP (|S| = *l*) and an edge is drawn between two SNP nodes, (*s*, *s*′), if *s* and *s*′ are covered together by at least one sequence fragment.

### 2.2. Haplotype Estimation of Tuberization Loci in Potato

We used PopPoly to estimate haplotypes of the *S. tuberosum* loci involved in tuber formation reported by Kloosterman et al. (2013), in an F1-population with 10 offspring obtained from the crossing of two *S. tuberosum* cultivars: Altus × Colomba (*A* × *C*). The nine investigated loci (Table 1) belong mainly to the potato cycling DOF factor (*StCDF*) gene family, but also include other genes, such as CONSTANS (CO) genes CO1 and CO2, that are shown to be involved in *StCDF* regulation (Kloosterman et al., 2013).

**Table 1:** *S. tuberosum* loci selected for haplotyping.

**Gene**	**DNA sequence id**	**Chromosome: coordinates**	**Segregating bi-allelic SNPs**
*StCDF1*	PGSC0003DMG400018408	chr05:4538880-4541736	38
*StCDF2*	PGSC0003DMG400025129	chr02:25588000-25591776	63
*StCDF3*	PGSC0003DMG400001330	chr02:46143998-46147444	75
*StCDF4*	PGSC0003DMG400033046	chr06:51598497-51601151	51
*StCDF5*	PGSC0003DMG400019528	chr03:55882564-55885296	100
*StCO1*	PGSC0003DMG401010056	chr02:45098374-45101578	57
*StCO2*	PGSC0003DMG402010056	chr02:45088023-45092647	66
*StFKF1*	PGSC0003DMG400019971	chr01:531784-536380	89
*StGI1*	PGSC0003DMG400001110	chr03:14265390-14266279	40

Sequence data for the parents and the offspring were obtained by whole genome sequencing (WGS) using Illumina HiSeq X Ten technology. Paired-end sequences were obtained with an average insert size of 380 bp (single read length of 151 bp) and aligned to PGSC-DM-v4.03 reference genome (Potato Genome Sequencing Consortium, 2011) using BWA-MEM (Li, 2013). Genomic variation within the boundaries of the selected genes was detected from the aligned reads using FreeBayes (Garrison and Marth, 2012), with an average read depth of 85× (sd=30×) at the target loci. The sequence and variant calling data were used by PopPoly to estimate the phasing of the detected bi-allelic SNP sites (including SNPs obtained by collapsing FreeBayes complex variants).

To evaluate the accuracy of the estimated haplotypes, we selected 9 haplotype tagging SNPs (*ht*SNPs) for the parents at the *StFKF1* locus (Supplement B), and obtained their genotypes by the KASP genotyping platform (Semagn et al., 2014). The reason for choosing this specific locus was that it had 8 distinct haplotypes which could be uniquely tagged by a subset of the SNPs in the locus far enough from their neighbor variants, so that the KASP primers could be properly designed. To choose the *ht*SNPs, we considered those SNPs whose dosages in combination were compatible with one and only one of the 36 possible transmissions of the parental haplotypes in the offspring, with some redundancy to still be able to tag the haplotypes in case of low genotyping quality for some of the SNPs.

Using the KASP assay, allele specific probe signals were obtained from the parents and 181 offspring from the *A* × *C* cross (including the 10 re-sequenced offspring). To determine the genotypes, we used the *R* package fitPoly (a modified version of the package fitTetra Voorrips et al., 2011), which clusters the probe signals using a mixture of normal distributions corresponding to the marker dosages, taking the segregation of parental alleles into account. The Pearson correlation coefficient between the KASP and PopPoly dosages at these *ht*SNPs was calculated in the parents and in the 10 resequenced offspring, as a measure of the overall similarity between the true and the estimated haplotypes.

## 3. Results

### 3.1. Simulation Study

To evaluate the performance of PopPoly, we simulated potato F1-populations with 1 to 30 offspring and estimated the population haplotypes using PopPoly as well as SDhaP (Das and Vikalo, 2015), H-PoP (Xie et al., 2016) and TriPoly (Motazedi et al., 2018a). The estimated haplotypes were compared to the original haplotypes by *hapcompare* (Motazedi et al., 2018b), using the measures introduced in section 2.1. The overall values for the haplotyping quality measures of each method, i.e., the average of each measure over offspring sizes from 1 to 30, are given in Table 2 and the main conclusions are summarized below.

**Table 2:** Average values and 95% confidence intervals for the quality measures of each haplotyping method, obtained by simulation at the sequencing depth of 5-5-2× (mother-father-offspring) per homolog.

	**PopPoly**	**TriPoly**	**H-PoP**	**SDhaP**
PAR	0.81(0.39;1)	0.71(0.35;1)	0.6(0.02;1)	0.44(0.04;0.93)
RR	0.95(0.8;1)	0.92(0.79;1)	0.89(0.7;1)	0.85(0.73;0.98)
SMR	0.1(0;0.33)	0.19(0;0.44)	0.33(0.04;0.64)	0.19(0;0.44)
IDR	0.09(0;0.31)	0.13(0;0.33)	0.2(0;0.69)	0.31(0;0.73)
NGPS	0.0009(0;0.001)	0.0009(0;0.001)	0.01(0;0.08)	0.01(0;0.08)

### 3.2. PopPoly Yields More Accurate Offspring Haplotypes

**Figure 2:**
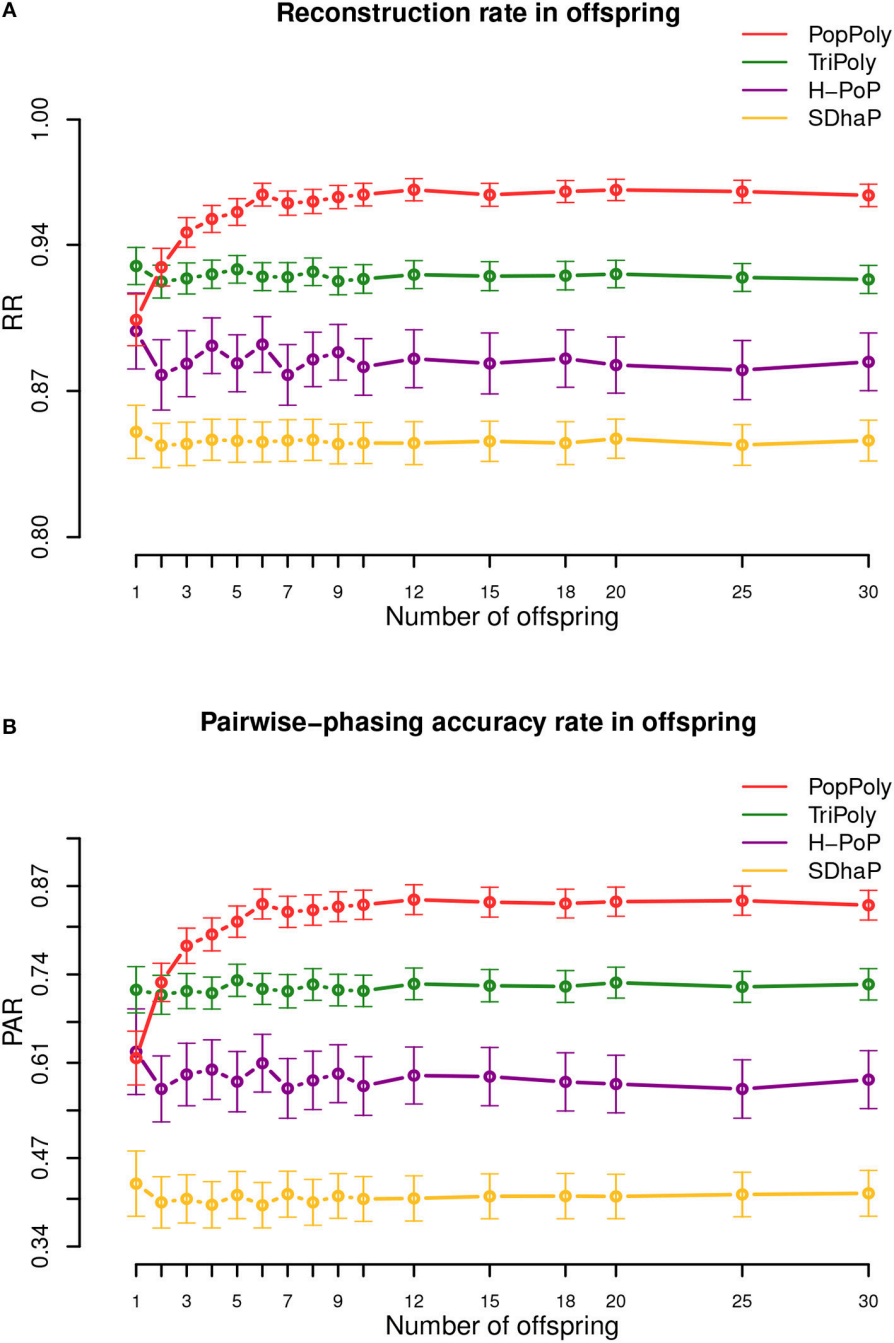
Haplotyping accuracy measures: **(A)** RR, **(B)** PAR in the offspring against the number of offspring in the population using PopPoly (red), TriPoly (green), H-PoP (purple), and SDhaP (yellow) for simulated tetraploid potato populations.

The average haplotype reconstruction rate (RR), which is a measure of overall phasing accuracy, obtained by PopPoly for the offspring was 0.96 (95% CI [0.87;1]) across different population sizes, which was higher than the other methods (Figure 2A). The second measure of accuracy, the pairwise-phasing accuracy rate (PAR) which is especially sensitive to the accuracy of phasing between distant SNPs, had an average value of 0.84 (95% CI [0.5;1]) by PopPoly for the offspring, which was the best among the applied methods (Figure 2B). The improvement in PAR using PopPoly was, however, more manifest compared to RR.

It was also noted that the accuracy of PopPoly depends on the population size, especially for distant phasing evaluated by PAR, although this dependence gradually diminishes as the number of offspring grows. As seen in Figure 2B, PAR increases rapidly for PopPoly with an increase in the number of offspring from 1 to 3 and in fact, the highest offspring score for a trio, i.e., with only one offspring, is reported by TriPoly. Since an increase in the count of each parental haplotype in the population, through an increase in the number of the offspring, results in an increase in the number of reads coming from each haplotype (assuming no sequencing bias), the power of the PopPoly algorithm to detect the parental haplotype is boosted with more offspring. With a tetraploid trio, however, there is a chance that some of the parental haplotypes are not transmitted to the offspring, which causes the lower accuracy of PopPoly compared to TriPoly.

For the parents, the reported accuracy measures were very similar between the methods. However, H-PoP and PopPoly yielded the highest scores (Figures S1, S2), with average PAR values of 0.64 (95% CI [0.2;1]) and 0.67 (95% CI [0;1]), and RR values of 0.89 (95% CI [0.73;1]) and 0.9 (95% CI [0.67;1]) for PopPoly and H-PoP, respectively.

While increasing the per homolog coverage from 5-5-2× (mother-father-offspring) to 30-30-30× yielded an average increase of 23-36% in PAR for TriPoly, H-PoP and SDhaP, the increase was only 14% for PopPoly (Figures S3–S5), as combining the population reads already effectively augments the haplotyping coverage (the increase was actually less than 5% with 10 offspring, Figure S5). Similarly, the difference in RR between the lowest and the highest coverage was 3% for PopPoly compared to 4-6% for the other methods (Figures S6–S8).

### 3.3. Haplotype Estimates of PopPoly Include More SNPs Than That of Other Methods

**Figure 3:**
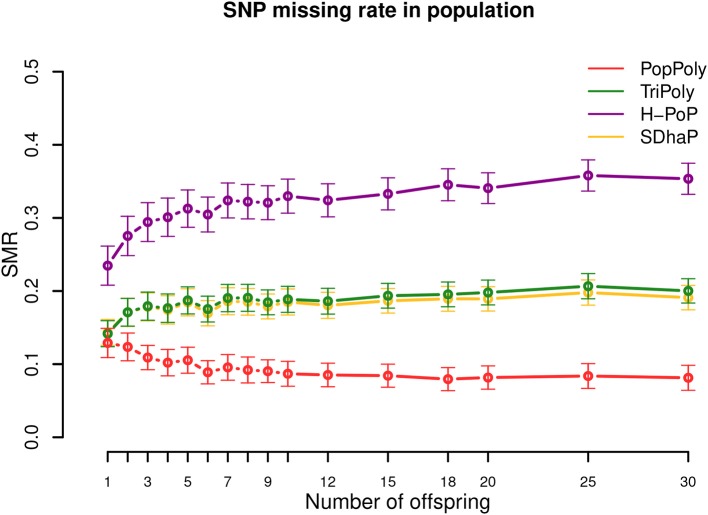
SNP missing rate (SMR) in the population against the number of offspring reported by PopPoly (red), TriPoly (green), H-PoP (purple), and SDhaP (yellow) for simulated tetraploid potato populations.

As seen in Table 2, the average SNP missing rate (SMR) of PopPoly was around 10%, which was 20% lower compared to H-PoP and around 10% lower compared to TriPoly and SDhaP (Figure 3). The reason for this is that combining individual NGS reads increases the chance to phase parental SNPs and choosing the offspring phasings from the estimated parental haplotypes leads to the inclusion of SNPs not sufficiently covered by the offspring reads, as well as to the imputation of SNPs uncalled in (some of) the offspring.

The 10% SMR of PopPoly can be explained by the algorithm's excluding a SNP position if the offspring genotypes at that position (either given as input or estimated anew) are incompatible with the surviving parental extensions. An example of this for a trio is the extension at *s* = 2, if the only surviving parental extensions are $H_{m}^{2}=H_{f}^{2}=\left(\begin{array}{ccccc} h_{1} & h_{2} & h_{3} & h_{4}\\ \text{s = 1:} & 0 & 0 & 1 & 1\\ \text{s = 2:} & 1 & 1 & 0 & 0 \end{array}\right)$ while the offspring genotypes at *s* = 1 and *s* = 2 are $G_{c}^{1}=H_{c}^{1}=\left(0,0,0,1\right)$ and $G_{c}^{2}=\left(1,1,1,1\right)$, respectively. In this case, $G_{c}^{2}$ is compatible with the parental genotypes at *s* = 2 (and therefore is accepted by the point-wise dosage estimation of PopPoly), but no $H_{c}^{2}$ can be obtained whose genotype at *s* = 2 is $G_{c}^{2}$, as haplotype $\left(\begin{array}{l} h_{c}\\ 1\\ 1 \end{array}\right)$ cannot be transmitted to the offspring without meiotic recombination in either $H_{m}^{2}$ or $H_{f}^{2}$. Since PopPoly is based on the assumption of no recombination (Appendix A in Supplementary Material), it excludes the SNP site *s* = 2 from phasing.

Increasing the per homolog sequencing depth from 5-5-2× (mother-father-offspring) to 30-30-30× decreased the SMR by 16–17% for SDhaP, PopPoly and TriPoly, and by 26% for H-PoP (Figures S9–S11).

### 3.4. PopPoly Improves SNP Dosage Estimation

**Figure 4:**
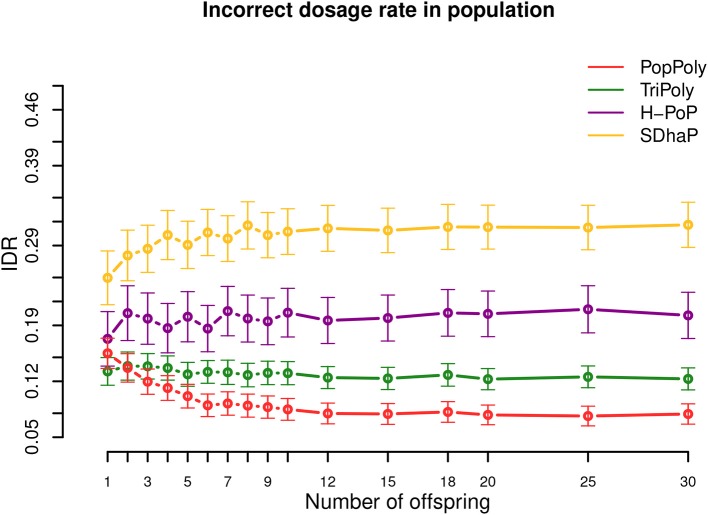
Incorrect dosage rate (IDR) in the population against the number of offspring reported by PopPoly (red), TriPoly (green), H-PoP (purple), and SDhaP (yellow) for simulated tetraploid potato populations.

As shown in Table 2 and Figure 4, among the haplotyping methods PopPoly yielded the lowest incorrect dosage rate (IDR) in the phased SNPs, which was 9% on average.

The differences in the IDR between the methods is due to the differences in each algorithm's approach to handle genotype dosages. Specifically, H-PoP attempts to obtain an optimal partitioning of the reads into *k* groups corresponding to the homologs of a *k*-ploid, so that the difference between the reads assigned to the same homolog is minimized and the difference between the reads assigned to different homologs is maximized. The haplotypes are determined by taking a consensus of the reads within each group, and the dosages are determined by the estimated haplotypes. SDhaP on the other hand employs a gradient descent scheme with Lagrangian relaxation to find the best phasing (in the space of all possible phasings) according to the MEC criterion. Thus, its MEC solution determines the dosages of the SNP alleles.

In contrast to H-PoP and SDhaP, TriPoly and PopPoly use the input dosages as basis and make corrections to these based on parent-offspring relationships in the population. Specifically, if the genotype of an offspring in a trio is not compatible with the genotypes of the parents at position *s*, TriPoly obtains the offspring extension and hence the offspring genotype at *s* by considering all of the possible allele transmissions from the parents at *s* and by choosing the most likely trio extensions. The dosage correction method of PopPoly is explained in Appendix B (Supplementary Material).

The simulation results show that the dosage correction scheme of PopPoly is the most successful approach if there are at least two offspring in the population (Figure 4). For a trio, however, the most accurate dosages are reported by TriPoly. As discussed for the phasing accuracy, the ability of PopPoly to detect wrongly estimated dosages and to correctly (re)estimate dosages depends on the haplotype counts in the population. Due to the absence of some parental haplotypes in the offspring of a trio, the accuracy of PopPoly drops below that of TriPoly, which relies less on the parental haplotypes and more on the reads of the offspring to assign its dosages. With at least 6 offspring, the IDR of PopPoly drops below 10% (~7%).

Considering the sequencing coverage, SDhaP profited the most from the higher depths with a 24% lower IDR at 30-30-30× compared to 5-5-2× (per homolog), while this decrease in IDR was 12% for TriPoly and H-PoP and only 7% for PopPoly (Figures S12–S14).

### 3.5. Continuity of Haplotyping Is Improved by PopPoly Compared to Single Individual Methods

**Figure 5:**
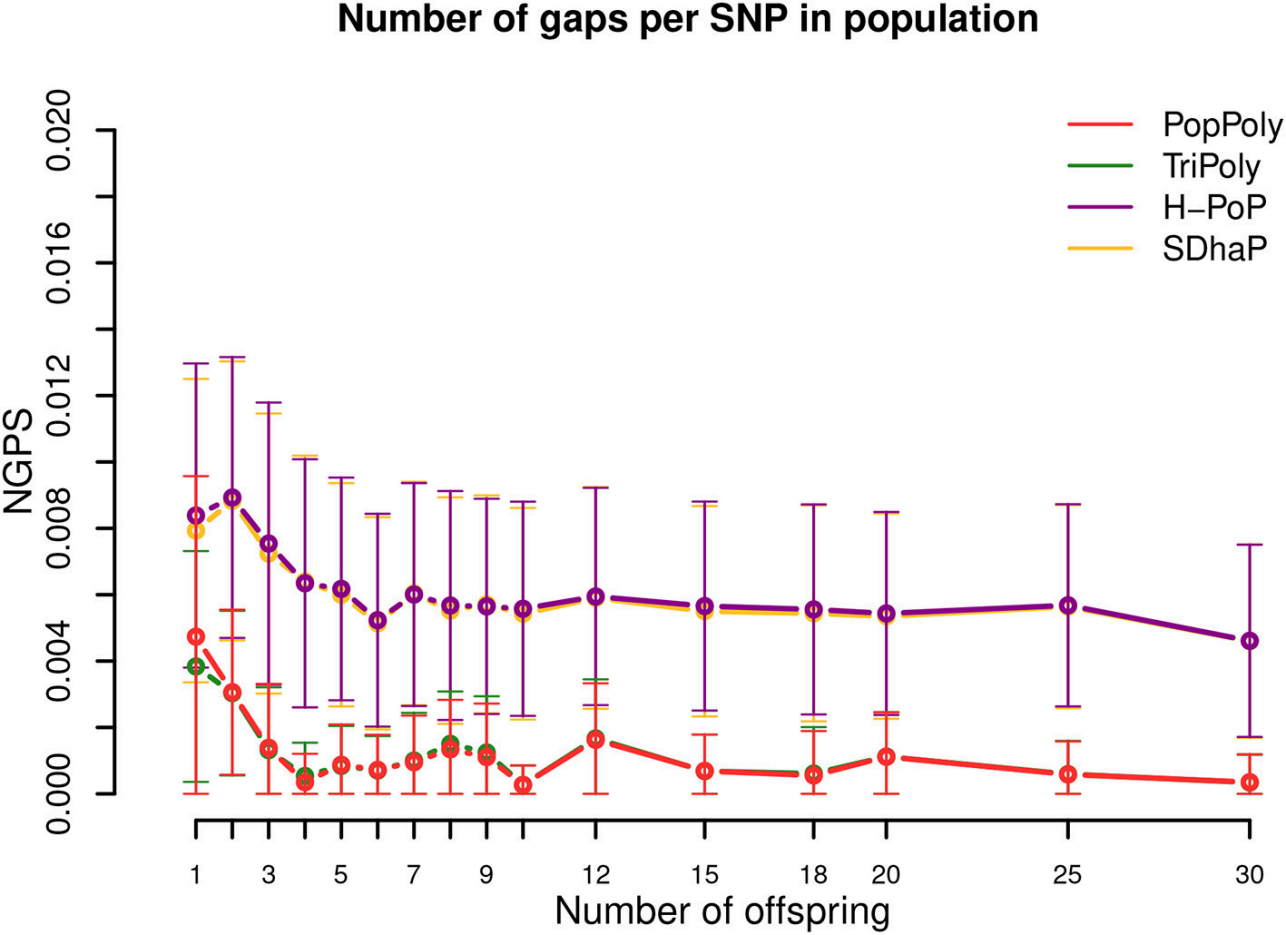
Number of phasing gaps normalized per SNP (NGPS) in the haplotype estimates of PopPoly (red), TriPoly (green), H-PoP (purple), and SDhaP (yellow) against the number of offspring in the population for simulated tetraploid potato populations.

As shown in Table 2 and Figure 5, the expected number of phasing gaps (normalized by the number of SNPs) is much lower in the estimates of TriPoly and PopPoly compared to H-PoP and SDhaP, as a pair of SNPs has a higher chance of being connected when all of the population reads are used for the phasing of each individual compared to the case where for each individual only its own reads are considered. Sequencing coverage was not a determining factor for this (Figures S15–S17).

### 3.6. Haplotypes of Tuberization Loci in the Tetraploid Potato Population

Using PopPoly, we phased all of the 579 segregating SNPs at 9 loci in the potato genome for a 10 offspring *A* × *C* cross (Supplement A). For each locus, we used the estimated haplotypes to calculate nucleotide diversity (Tajima, 1983), i.e., the expected chance of a nucleotide difference per site between two randomly chosen haplotypes in the population. While the rather low nucleotide diversity values at the loci (mean=0.37, *SD* = 0.06) showed high local similarity between the haplotypes, the numbers of distinct haplotypes were rather high, at 5 loci equal to the maximum of 8 (Table 3).

**Table 3:** Summary of SNP phasing at the potato loci introduced in Table 1.

**Gene**	**Number of distinct parental haplotypes**	**Transmission counts of parental haplotypes^⋇^**	**Nucleotide diversity**
*StCDF1*	6	4-5-15	0.40
*StCDF2*	8	2-4.5-8	0.43
*StCDF3*	8	1-5-9	0.28
*StCDF4*	3	7-15-18	0.42
*StCDF5*	7	1-5-10	0.32
*StCO1*	3	8-11-21	0.40
*StCO2*	8	1-5-10	0.41
*StFKF1*	8	2-5-8	0.38
*StGI1*	8	1-4.5-9	0.29

⋇ *Minimum-Median-Maximum count of the distinct parental haplotypes observed in the offspring*.

As evident from the median counts of the transmission of parental haplotypes to the offspring in Table 3, around half of the 58 distinct parental haplotypes (over all of the loci) were transmitted at least 5 times to the offspring. This is the expected transmission count of a haplotype in a tetraploid cross with 10 offspring if all of the parental haplotypes are distinct at the locus. However, larger sample sizes are needed to formally test whether the transmission patterns of the haplotypes are as expected under random segregation (Appendix A in Supplementary Material).

### 3.7. Validation of PopPoly Estimated Haplotypes

Based on the *ht*SNPs, a KASP assay was designed to investigate the eight distinct parental haplotypes of the *StFKF1* locus (Supplement B). We checked the segregation of the parental haplotypes using the *ht*SNPs at this locus in 181 offspring of the *A* × *C* cross, including the 10 sequenced offspring previously used in the estimation of the haplotypes with PopPoly. The obtained KASP signal ratios and the genotypes estimated by fitPoly are given in Supplement C. The KASP data was used to 1) calculate the correlation between the *ht*SNP dosages estimated from the whole genome sequencing data and the KASP dosages and 2) assess the transmission of the eight haplotypes in the 181 offspring individuals according to genetic rules, i.e., the expected transmission ratio of each maternal and paternal haplotype.

A correlation of 0.94 was observed in the comparison between the dosages of the *ht*SNPs observed in the sequencing data and the KASP data (varying within the range 0.85–1 per individual), in the 10 offspring assessed with both technologies (Figure S18). As the SNP dosages are estimated by fitting a probabilistic model for both the sequencing and the KASP assay, both approaches are prone to estimation error. The differences between the called dosages can also hinder choosing the transmitted parental haplotypes for the offspring in the larger KASP genotyped population. Therefore, some inconsistencies between the chosen haplotypes for each offspring and its KASP dosages are to be expected.

Within the larger KASP genotyped offspring population, we could assess the transmission counts of the eight haplotypes for *StFKF1* locus using genetic rules. 92% of the 181 offspring could be unambiguously phased, each consisting of two haplotypes from each parent. The 8% failure rate in uniquely choosing the haplotypes could be mainly attributed to the non-calling rate of around 2% observed for the *ht*SNPs in these individuals, as well as to inconsistencies between the dosage estimates of the *ht*SNPs obtained by the KASP assay and by PopPoly. As mentioned above, we therefore had to allow for some difference between the haplotypes and the KASP genotypes. Specifically, for each offspring we chose from the 36 possible parental transmissions the phasing that had the highest match in terms of the SNP dosages with its KASP genotypes (after eliminating SNP number 5, which had a very high inconsistency rate and was also redundant for tagging).

Subsequently, we assessed the consistency of the uniquely estimated phasings with the assumptions of random polysomic segregation. For this purpose, χ^2^ goodness-of-fit tests were performed for the transmission of each haplotype from each parent, which showed no significant deviation at α = 0.05. This suggests that the PopPoly prediction for each of the eight *StFKF*1 haplotypes is correct. However, the obtained results also show that accurate SNP dosage calling is challenging in polyploids.

## 4. Conclusion and Discussion

We present a novel algorithm, PopPoly, to exploit parent-offspring relationships for the estimation of haplotypes in an outcrossing F1-population that consists of two heterozygous parents and their F1 offspring, using short DNA sequence reads and SNP genotypes called in the population. In this approach, we first estimate the phasings of the parents by combining the sequence reads of the whole population. If necessary, SNP genotypes are also (re)estimated for the parents from the reads considering parent-offspring relationships. Having the parental phasings, we determine the phasing of each offspring by choosing from the possible transmissions of the parental haplotypes, such that the phasing chosen for each offspring has maximal compatibility with its individual reads. A natural advantage of obtaining offspring phasings from the parents is that the SNP genotypes uncalled in an offspring are imputed in its haplotypes, provided that these SNPs are included in the parental phasings.

The polyploid haplotyping problem is NP-hard and practical solutions thus by necessity depend on approximate optimization methods. PopPoly takes a greedy approach based on Bayesian probability, extending haplotype estimates one position at a time starting from the leftmost position. While PopPoly is similar in this respect to TriPoly (Motazedi et al., 2018a), its underlying model is quite different. As such, PopPoly is to our knowledge the first method that uses the information of siblings in estimating the haplotypes of each offspring.

Through simulations, we showed that PopPoly outperforms single individual haplotyping methods, which ignore family relationships. Besides, PopPoly yields better estimates compared to the trio based haplotyping method TriPoly when there are more than 2 offspring in the population. In addition, PopPoly uses Mendelian segregation to improve variant dosage estimation in the population at the detected SNP sites. We also show that the performance of PopPoly is influenced less by sequencing depth than competing methods. While PopPoly assumes no limitation on the size of the population, computational resources become an important limitation when the number of offspring exceeds a couple hundred, which might require the division of a large population into smaller subpopulations for phasing. Also, the probability of observing recombinations in the F1 generation increases as the number of offspring grows. However, with genomic regions that are often at most 20 *kb* long, a typical maximum range for short read haplotyping, at least 500 offspring are needed to expect 1 recombination event in potato F1 populations, even at relatively high recombination rates of around 8 *cM*/*Mb*. This is not expected to have a substantial impact on the accuracy of the estimates of the parents and the other offspring.

To demonstrate the utility of PopPoly, we used it to phase 579 SNPs segregating at 9 tuberization loci in an F1 population of tetraploid potato, the *A* × *C* cross, with 10 offspring. Using the KASP assay genotypes of a set of *ht*SNPs to represent the true haplotypes, we found a high correlation between the PopPoly estimates and the true haplotypes in the *A* × *C* population. We were able to uniquely determine the haplotypes at the tagged locus with a 92% success rate, using the parental haplotypes estimated by PopPoly and the KASP genotypes at the *ht*SNPs in 171 offspring of the *A* × *C* cross that had not been sequenced. We demonstrated that by sequencing the parents and a few offspring one can obtain the set of population (or family) haplotypes, from which the haplotypes of each individual can be determined using a set of genotyped *ht*SNPs. Such a strategy can be suitably adopted in QTL studies, with typical sizes of a few hundreds to a few thousands individuals, to increase the statistical power and to ease the interpretation of results.

## Software

PopPoly was developed in Python 2.7.0 and is freely available on the software page of the Bioinformatics group, Wageningen University and Research: http://www.bif.wur.nl.

## Author Contributions

EM developed the methods and wrote the manuscript. All authors contributed to the design of the study and to the manuscript, and approved its final version.

### Conflict of Interest Statement

The authors declare that the research was conducted in the absence of any commercial or financial relationships that could be construed as a potential conflict of interest.
