# Supplementary material for: Family-Based Haplotype Estimation and Allele Dosage Correction for Polyploids Using Short Sequence Reads
Source: Front Genet. 2019 Apr 16;10:335. doi: 10.3389/fgene.2019.00335 (PMC6477055; doi:10.3389/fgene.2019.00335)
Supplement: Supplementary file 4 [file Data_Sheet_4.pdf]

APPENDICES: FAMILY-BASED HAPLOTYPE ESTIMATION AND ALLELE DOSAGE CORRECTION FOR POLYPLOIDS USING SHORT SEQUENCE READS

Appendix A

Estimation of parental haplotypes

Inspired by the approach of Berger *et al.* (2014), we start at the first SNP position in the target region ($s = 1$), and extend the maternal and paternal genotypes of this SNP, $G_m^1 = H_m^1$ and $G_f^1 = H_f^1$, respectively, to two-SNP phasings, H_m^2 and H_f^2. We consider every possible phasing between H_m^1 and H_f^1 and SNP position $s = 2$ in the region, and obtain the joint conditional probability of each extension pair, (H_m^s, H_f^s), at $s = 2$ given the sequence reads of the population and the parental genotypes, (G_m^s, G_f^s), as well as the offspring genotypes $G_{c_i}^s$ for $i = 1, \dots, n$ (with n representing the number of offspring). Keeping only those parental extensions whose conditional probability exceeds or equals a pre-set *branching* threshold, $\rho \in (0, 1]$, we eliminate further the extensions whose probability is less than κP_{max}, where $\kappa \in [0, 1]$ is a pre-set *pruning* threshold and P_{max} is the maximum probability assigned to the candidate parental extensions. The surviving extensions at $s = 2$ are used in the next step as base phasings to obtain the extensions at $s = 3$ in a similar manner, and this procedure is iterated until the last SNP $s = l$ has been added to the parental extensions.

As it is not straightforward to directly calculate the conditional extension probabilities (Motazedi *et al.*, 2018), we calculate instead the probability of the sequence reads conditional on each possible phasing and convert these probabilities to the desired extension probabilities using Bayes' formula:

$$P(H_m^s, H_f^s | H_m^{s-1}, H_f^{s-1}, G_m^s, G_f^s, G_{c_1}^s, \dots, G_{c_n}^s, \mathbf{R}_{set}, \boldsymbol{\epsilon}_{set}) = \frac{P(\mathbf{R}_{set} | H_m^s, H_f^s, \boldsymbol{\epsilon}_{set}) P(H_m^s, H_f^s | G_m^s, G_f^s, G_{c_1}^s, \dots, G_{c_n}^s, H_m^{s-1}, H_f^{s-1})}{\sum\limits_{(H_m^s, H_f^s)'} P\big(\mathbf{R}_{set} | (H_m^s, H_f^s)', \boldsymbol{\epsilon}_{set}\big) P\big((H_m^s, H_f^s)' | G_m^s, G_f^s, G_{c_1}^s, \dots, G_{c_n}^s, H_m^{s-1}, H_f^{s-1}\big)} \tag{1}$$

where $\mathbf{R}_{set}$ denotes the set of all of the reads in the population and $\boldsymbol{\epsilon}_{set}$ stands for the set of base-calling error vectors, ϵ_j, associated with each $r_j \in \mathbf{R}_{set}$ ($1 \leqslant j \leqslant |\mathbf{R}_{set}|$). $P(\mathbf{R}_{set} | H_m^s, H_f^s, \boldsymbol{\epsilon}_{set})$ denotes the conditional probability of observing the reads given a pair of maternal and paternal extensions at s, (H_m^s, H_f^s), and the base-calling error probabilities given by $\boldsymbol{\epsilon}_{set}$.

To calculate $P(\mathbf{R}_{set} | H_m^s, H_f^s, \boldsymbol{\epsilon}_{set})$, we assume conditional independence of each read, $r_j \in \mathbf{R}_{set}$, from the other reads in $\mathbf{R}_{set}$ given $\boldsymbol{\epsilon}_{set}$, and use the fact that each read is either directly obtained from one of the parental samples or belongs to an offspring c_i ($i = 1, ..., n$), in which latter case the read may have originated from either parent with equal probability. Under these assumptions, $P(\mathbf{R}_{set} | H_m^s, H_f^s, \boldsymbol{\epsilon}_{set})$ is determined according to:

$$P(\mathbf{R}_{set}|H_m^s, H_f^s, \boldsymbol{\epsilon}_{set}) = \prod_{j=1}^{|\mathbf{R}_{set}|} P(r_j|H_m^s, H_f^s, \boldsymbol{\epsilon}_{set}) =$$

$$\prod_{j=1}^{|\mathbf{R}_{set}|} \Big[P(r_j|H_m^s, \epsilon_j) U\big(\delta(r_j), m\big) + P(r_j|H_f^s, \epsilon_j) U\big(\delta(r_j), f\big) + \tag{2}$$

$$\frac{1}{2}\big(P(r_j|H_m^s, \epsilon_j) + P(r_j|H_f^s, \epsilon_j)\big) \sum_{i=1}^{n} U\big(\delta(r_j), c_i\big)\Big]$$

$$U(x, y) = \begin{cases} 1 & x = y \\ 0 & x \neq y \end{cases}$$

$$\delta : \mathbf{R}_{set} \longrightarrow \{m, f, c_1, ..., c_n\}$$

where the function $\delta(r_j)$ returns the origin of read r_j: mother (m), father (f), or one of the n offspring ($c_1, ..., c_n$).

Assuming independence of the sequencing errors at the SNP positions within each read, $P(r_j|H_m^s)$ and $P(r_j|H_f^s)$ in Equation 2 can be calculated according to Motazedi *et al.* (2018):

$$P(r_j|H_p^s, \epsilon_j) = \frac{1}{k_t} \sum_{h \in H_p^s} P(r_j|h, \epsilon_j) \qquad p \in \{m, f\}$$

$$P(r_j|h, \epsilon_j) = \prod_{\tau=1}^{s} \frac{1}{3}\epsilon_j^\tau d(r_j, h, \tau) + \frac{1 - \epsilon_j^\tau}{1 - \frac{2}{3}\epsilon_j^\tau}\big(1 - d(r_j, h, \tau)\big) \tag{3}$$

$$d(r_j, h, \tau) = \begin{cases} 1 & r_j^\tau \neq h^\tau, r_j^\tau \neq \text{"-"}, h^\tau \neq \text{"-"} \\ 0 & otherwise \end{cases}$$

where ϵ_j assigns a base-calling error probability to every SNP position in r_j, and h stands for each of the k_t homologues in the phasing extension H_p^s ($p \in \{m, f\}$). In Equation 3, we use the superscript τ in r_j^τ and ϵ_j^τ to represent the called base at SNP position τ and its associated error probability, respectively. Likewise, h^τ denotes the allele assigned to homologue h at SNP position τ. We use r_j^τ = "-" and h^τ = "-" to show that SNP position τ has not been called in r_j or is missing in h.

In obtaining $P(r_j|h, \epsilon_j)$ in Equation 3, we assume that an erroneously called base can with equal chance be any of the three wrong bases. Therefore, the probability of observing a specific wrong allele is $\frac{1}{3}\epsilon_j^\tau$. Also, the probability of no error is actually the probability that no error occurs $(1-\epsilon_j^\tau)$, conditional on having observed either the reference or the alternative allele $(1 - \frac{2}{3}\epsilon_j^\tau)$. Therefore, it is $\frac{1-\epsilon_j^\tau}{1-\frac{2}{3}\epsilon_j^\tau}$.

Equations 2 and 3 establish the procedure to calculate the likelihood in Bayes' formula in Equation 1. In order to solve Equation 1, one also needs to specify the prior, $P(H_m^s, H_f^s|G_m^s, G_f^s, G_{c_1}^s, \ldots, G_{c_n}^s, H_m^{s-1}, H_f^{s-1})$. While several ways can be thought of to specify this prior, we obtain it as follows. As the parental extensions (H_m^s, H_f^s) are confined to those compatible with G_m^s and G_f^s, we set this prior to zero for every incompatible extension. For the

compatible extensions, we look into the possible transmissions of the extended haplotypes (ignoring phenomena like aneuploidy (Karp *et al.*, 1982), preferential chromosome pairing (Bourke *et al.*, 2017), recombination and double reduction (Bourke *et al.*, 2015)) to the offspring and for each offspring, c_i, we count the number of transmissions that agree with its genotype at s, $G^s_{c_i}$. Dividing this number by the total number of possible transmissions, $\binom{k_m}{\frac{k_m}{2}} \cdot \binom{k_f}{\frac{k_f}{2}}$, gives us $P(G^s_{c_i}|H^s_m, H^s_f)$. Calculating $P(G^s_{c_i}|H^s_m, H^s_f)$ for $i = 1, \ldots, n$, we obtain the average likelihood of an *observed* offspring genotype according to:

$$E_{H^s_m,H^s_f}[P(G^s_c|H^s_m, H^s_f)] = \sum_{i=1}^{n} \frac{P(G^s_{c_i}|H^s_m,H^s_f)}{P(G_{c_1}|H^s_m,H^s_f)+\cdots+P(G_{c_n}|H^s_m,H^s_f)} P(G^s_{c_i}|H^s_m, H^s_f)$$
$$= \frac{1}{\sum\limits_{i=1}^{n} P(G_{c_i}|H^s_m,H^s_f)} \sum_{i=1}^{n} \left(P(G^s_{c_i}|H^s_m, H^s_f)\right)^2 \quad (4)$$

where $P(G^s_{c_i}|H^s_m, H^s_f)$ is the likelihood and $\frac{P(G^s_{c_i}|H^s_m,H^s_f)}{P(G_{c_1}|H^s_m,H^s_f)+\cdots+P(G_{c_n}|H^s_m,H^s_f)}$ is the probability of observing offspring c_i.

So far, we set the prior for each (H^s_m, H^s_f) to be proportional to $E_{H^s_m,H^s_f}[P(G^s_c|H^s_m, H^s_f)]$. However, as changing the order of the homologues does not change a phasing, several permutations of the alleles at $s-1$ and s can yield the same (H^s_m, H^s_f). Therefore, the prior should also be proportional to the number of permutations that result in (H^s_m, H^s_f). It can be thus set to:

$$P(H^s_m, H^s_f|G^s_m, G^s_f, G^s_{c_1}, \ldots, G^s_{c_n}, H^{s-1}_m, H^{s-1}_f) = E_{H^s_m,H^s_f}[P(G^s_c|H^s_m, H^s_f)] \frac{\binom{k_m!}{\omega^{sm}_1! \ldots \omega^{sm}_{u_m}!}}{\Pi^m_{s-1}\Pi^m_s} \frac{\binom{k_f!}{\omega^{sf}_1! \ldots \omega^{sf}_{u_f}!}}{\Pi^f_{s-1}\Pi^f_s} \quad (5)$$

where, for $p \in \{m, f\}$, Π^p_{s-1} and Π^p_s are the number of possible permutations of the alleles at $s-1$ and s, respectively, u_p is the number of distinct homologues, i.e. haplotypes, in H^s_p regarding only positions $s-1$ and s, and ω^{sp}_i for $i \in \{1, ..., u_p\}$ denotes the number of times an identical haplotype (regarding only positions $s-1$ and s) is present in H^s_p. Although it is possible to normalise the priors obtained this way over all of the possible extensions (to obtain a proper prior mass function), one does not need to do so as the discrete posteriors are normalised anyway at the end.

As an example, with tetraploid parents there will be $\binom{4}{2} \cdot \binom{4}{2} = 36$ possible haplotype transmissions to each offspring. With maternal and paternal extensions at $s = 3$ being equal to $H^3_m = \begin{pmatrix} & h_1 & h_2 & h_3 & h_4 \\ \text{SNP 1:} & 1 & 1 & 0 & 0 \\ \text{SNP 2:} & 1 & 0 & 0 & 1 \\ \text{SNP 3:} & 1 & 0 & 1 & 1 \end{pmatrix}$ and $H^3_f = \begin{pmatrix} & h_5 & h_6 & h_7 & h_8 \\ \text{SNP 1:} & 0 & 1 & 0 & 0 \\ \text{SNP 2:} & 0 & 0 & 1 & 1 \\ \text{SNP 3:} & 0 & 0 & 0 & 1 \end{pmatrix}$, respectively, and two offspring c_1 and c_2 with $G^3_{c_1} = (1\,0\,0\,0)$ and $G^3_{c_2} = (1\,0\,1\,0)$, only 9 out of 36 transmissions will be compatible with the genotype of c_1, while 18 transmissions will be compatible with c_2. This results in $E_{H^s_m,H^s_f}[P(G^3_c|H^3_m, H^3_f)] = \frac{1}{\frac{3}{4}}\left((\frac{9}{36})^2 + (\frac{18}{36})^2\right) = \frac{5}{12}$ for this extension. As $k_m = k_f = 4$, $G^2_m = (1, 0, 0, 1)$, $G^3_m = (1, 0, 1, 1)$, $G^2_f = (0, 0, 1, 1)$ and $G^3_f = (0, 0, 0, 1)$, we have $\Pi^m_2 = \Pi^f_2 = \binom{4!}{2!2!} = 6$ and $\Pi^m_3 = \Pi^f_3 = \binom{4!}{3!1!} = 4$. Considering only SNPs at $s-1 = 2$ and $s = 3$,

in each parent there is one haplotype present twice. The a priori probability of (H_m^3, H_f^3) is hence determined from Equation 5 to be $\frac{5}{12} \cdot \frac{\binom{4!}{2!1!1!}}{24} \cdot \frac{\binom{4!}{2!1!1!}}{24} = \frac{5}{48}$.

From Equations 2 and 5, the conditional probabilities of parental extensions at position s can be obtained using Equation 1 and the surviving extensions are used for the extension to $s+1$, as explained above.

Appendix B

Estimation of missing and erroneous genotypes

The SNP-by-SNP extension of the parental haplotypes using the sequencing reads of an F1-population is explained in Appendix A, assuming the SNPs have been accurately called for all of the population members. However, in practice every haplotyping algorithm has to handle missing and wrongly estimated SNP genotypes caused by sequencing and variant calling errors.

In presence of wrongly estimated genotypes (wrong dosages), it can occur that all of the offspring genotypes are incompatible with the parental extensions at some SNP position s. At these positions, the extension should either be skipped, as the prior weight of all candidate phasings will be zero, or the genotypes must be estimated anew. The extension at s will also be impossible if one or both of the parental genotypes are missing at s. To include these SNP positions in the extension, it is necessary to impute the missing genotypes.

In order to estimate the population genotypes at the missing or incompatible positions, we assume that the parents come from an infinite-size population at Hardy-Weinberg equilibrium. Limiting the attention to bi-allelic SNPs, the reference and alternative allele frequencies of the parents at position s can be estimated from the observed reads under the above assumption. Assuming a fixed sequencing error rate for all of the reads and nucleotide positions, $0 \leqslant \widehat{ER} < 0.5$, the frequency of the alternative allele can be obtained assuming a binomial model for the observed count of the alternative allele according to:

$$\begin{aligned} \xi &= |\{r_j \in \mathbf{R}_{set} | r_j^s = 1 \vee r_j^s = 0\}| \\ \psi &= \frac{|\{r_j \in \mathbf{R}_{set} | r_j^s = 1\}|}{\xi} \\ \hat{p} &= \frac{\psi - \widehat{ER}}{1 - 2\widehat{ER}} \end{aligned} \tag{6}$$

where ξ is the total sequencing coverage of the population at s and ψ is the proportion of the alternative allele among the observed alleles. As this observed frequency, ψ, depends on the latent true frequency, $\hat{p}$, through $\psi = (1-\widehat{ER})\hat{p} + \widehat{ER}(1-\hat{p})$, it is straightforward to show that $\hat{p}$ can be obtained as shown in Equation 6, with a standard error equal to $\frac{1}{(1-2\widehat{ER})} \cdot \sqrt{\frac{\psi(1-\psi)}{\xi}}$.

In case a specific base-calling error rate ϵ_j^s is assigned at each position s to each read r_j, e.g. by using the integer-rounded Phred (quality) scores reported by the sequencer (Edgar and Flyvbjerg, 2015), one can assume a Gaussian distribution for the probability of observing the alternative allele at s in each read, $f_s\big(P(r_j)|\hat{p}, \hat{\sigma}^2\big) = \frac{1}{\sqrt{2\pi\hat{\sigma}^2}} e^{-\frac{(P(r_j)-\hat{p})^2}{2\hat{\sigma}^2}}$, and obtain $\hat{p}$ at each s according to:

$$\hat{p} = \frac{\displaystyle\sum_{\{r_j \in \mathbf{R}_{set} | r_j^s = 1 \vee r_j^s = 0\}} P(r_j)}{\xi} \tag{7}$$

$$\hat{\sigma}^2 = \frac{\sum (P(r_j) - \hat{p})^2}{\xi - 1}$$

$$P(r_j) = (1 - \epsilon_j^s) r_j^s + \epsilon_j^s (1 - r_j^s)$$

Having $\hat{p}$, a prior probability can be assigned to each of the 2^{k_m} and 2^{k_f} theoretically possible genotypes for the mother and the father, respectively, assuming a binomial model according to:

$$P(G_p^s) = \binom{k_t}{\nu} \hat{p}^{\nu} (1 - \hat{p})^{(k_t - \nu)} \tag{8}$$

where $p \in \{m, f\}$ and $0 \leqslant \nu \leqslant k_t$ is the dosage of the alternative allele in the candidate genotype, G_p^s. Assuming the parents have been independently chosen from a source population, a prior can be assigned to each (G_m^s, G_f^s) pair using $P(G_p^s)$ obtained from Equation 8, according to:

$$P(G_m^s, G_f^s) = P(G_m^s) \cdot P(G_f^s) \tag{9}$$

Given (G_m^s, G_f^s), a prior probability can be assigned to each specific offspring genotype, $G_{c_i}^s$, by counting the number of allele transmissions that result in that $G_{c_i}^s$. For example, with $(G_m^s, G_f^s) = \big((0,1,1,1),(1,0,0,0)\big)$, the prior $P(G_{c_1} | G_m^s, G_f^s)$ will be equal to 0, $\frac{9}{\binom{4}{2}\binom{4}{2}} = \frac{1}{4}$, $\frac{18}{\binom{4}{2}\binom{4}{2}} = \frac{1}{2}$, $\frac{9}{\binom{4}{2}\binom{4}{2}} = \frac{1}{4}$ and 0 for the offspring genotypes: $G_{c_1} = (0,0,0,0)$, $G_{c_1} = (1,0,0,0)$, $G_{c_1} = (1,1,0,0)$, $G_{c_1} = (1,1,1,0)$ and $G_{c_1} = (1,1,1,1)$, respectively.

To estimate the population genotypes, $(G_m^s, G_f^s, G_{c_1}^s, \cdots, G_{c_n}^s)$, we use the prior probabilities obtained as explained above, and assign a posterior probability to each population genotype by taking the sequencing reads into account. Noting that:

$$P(G_m^s, G_f^s, G_{c_1}^s, \cdots, G_{c_n}^s | \mathbf{R}_{set}, \boldsymbol{\epsilon}_{set}) = P(G_{c_1}^s, \cdots, G_{c_n}^s | G_m^s, G_f^s, \mathbf{R}_{set}, \boldsymbol{\epsilon}_{set}) P(G_m^s, G_f^s | \mathbf{R}_{set}, \boldsymbol{\epsilon}_{set}) \tag{10}$$

we separately obtain the posterior of the parental genotypes, $P(G_m^s, G_f^s | \mathbf{R}_{set}, \boldsymbol{\epsilon}_{set})$, and the conditional posterior of the offspring $P(G_{c_1}^s, \cdots, G_{c_n}^s | G_m^s, G_f^s, \mathbf{R}_{set}, \boldsymbol{\epsilon}_{set})$, from which the population posterior is derived using Equation 10. The posterior $P(G_m^s, G_f^s | \mathbf{R}_{set}, \boldsymbol{\epsilon}_{set})$ can be directly obtained from Equations 1 and 2 by substituting (H_m^s, H_f^s) with (G_m^s, G_f^s) in these equations and by using $P(G_m^s, G_f^s)$ (obtained by Equation 9) as the prior in Equation 1. Assuming conditional independence of the offspring genotypes given the parents, we obtain $P(G_{c_1}^s, \cdots, G_{c_n}^s | G_m^s, G_f^s, \mathbf{R}_{set}, \boldsymbol{\epsilon}_{set})$ by:

$$P(G^s_{c_1}, \cdots, G^s_{c_n} | G^s_m, G^s_f, \mathbf{R}_{set}, \boldsymbol{\epsilon}_{set}) = P(G_{c_1} | G^s_m, G^s_f, \mathbf{R}_{c_1}, \boldsymbol{\epsilon}_{c_1}) \cdot \ldots \cdot P(G_{c_n} | G^s_m, G^s_f, \mathbf{R}_{c_n}, \boldsymbol{\epsilon}_{c_n})$$

$$\mathbf{R}_{c_i} = \{r_j \in \mathbf{R}_{set} | \; \delta(r_j) = c_i\} \tag{11}$$

$$\boldsymbol{\epsilon}_{c_i} = \{\epsilon_j \in \boldsymbol{\epsilon}_{set} | \; \delta(r_j) = c_i\}$$

where $P(G_{c_i} | G^s_m, G^s_f, \mathbf{R}_{c_i}, \boldsymbol{\epsilon}_{c_i})$ is calculated according to:

$$P(G_{c_i} | G^s_m, G^s_f, \mathbf{R}_{c_i}, \boldsymbol{\epsilon}_{c_i}) = \frac{P(\mathbf{R}_{c_i} | G^s_{c_i}, \boldsymbol{\epsilon}_{c_i}) P(G^s_{c_i} | G^s_m, G^s_f)}{\sum\limits_{G'^s_{c_i}} P(\mathbf{R}_{c_i} | G'^s_{c_i}, \boldsymbol{\epsilon}_{c_i}) P(G'^s_{c_i} | G^s_m, G^s_f)} \tag{12}$$

and:

$$P(\mathbf{R}_{c_i} | G^s_{c_i}, \boldsymbol{\epsilon}_{c_i}) = \prod_{(r_j, \epsilon_j) \, \in \, \mathbf{R}_{c_i} \times \boldsymbol{\epsilon}_{c_i}} P(r_j | G^s_{c_i}, \epsilon_j) \tag{13}$$

where $\mathbf{R}_{c_i} \times \boldsymbol{\epsilon}_{c_i}$ represents the Cartesian product of $\mathbf{R}_{c_i}$ and $\boldsymbol{\epsilon}_{c_i}$, and (r_j, ϵ_j) denotes $r_j \in \mathbf{R}_{c_i}$ with its matched error rate vector, $\epsilon_j \in \boldsymbol{\epsilon}_{c_i}$. In Equation 13, $P(r_j | G^s_{c_i}, \epsilon_j)$ is obtained by replacing H^s_p with $G^s_{c_i}$ in Equation 3.

After calculating $P(G^s_m, G^s_f, G^s_{c_1}, \cdots, G^s_{c_n} | \; \mathbf{R}_{set}, \boldsymbol{\epsilon}_{set})$ from Equation 10, the most likely population genotypes at s can be assigned to the population members as genotype estimates.

Appendix C

Estimation of the offspring haplotypes

Having the set of all possible offspring phasings obtained by the possible transmissions of the parental haplotypes (Appendix A), we assign to each offspring c_i the phasing estimate $\hat{H}_{c_i}$ that yields the smallest number of required base-calling changes in the sequence reads, $\mathbf{R}_{c_i}$, in order to assign each $r_j \in \mathbf{R}_{c_i}$ to some homologue in $\hat{H}_{c_i}$. For each possible offspring phasing, $\hat{H}$, this required number of base-calling changes equals the so-called *minimum error correction* (*MEC*) score, defined as (Lippert *et al.*, 2002):

$$\mathit{MEC}(\hat{H}, \mathbf{R}_{c_i}) = \sum_{r_j \, \in \, \mathbf{R}_{c_i}} \min_{\hat{h} \in \hat{H}} D(r_j, \hat{h}) \tag{14}$$

$D(r_j, \hat{h})$ is the Hamming distance between read $r_j \in \mathbf{R}_{c_i}$ and homologue $\hat{h} \in \hat{H}$ defined according to:

$$D(r_j, \hat{h}) = \sum_{\tau=1}^{l} d(r_j, \hat{h}, \tau) \tag{15}$$

where τ and l represent the SNP positions and the number of SNPs in the target region, respectively, and $d(r_j, \hat{h}, \tau)$ is defined in Equation 3. Thus, for each c_i we have $\hat{H}_{c_i} = \underset{\hat{H}}{\operatorname{argmin}} \, \mathit{MEC}(\hat{H}, \mathbf{R}_{c_i})$. If $\hat{H}_{c_i}$ is the same as the true phasing of c_i, its MEC score is expected to be close to the number of actual base-call errors in $\mathbf{R}_{c_i}$.

In case more than one set of parental haplotypes has the maximum probability (Appendix A), we infer the offspring haplotypes for each of them as explained above and finally choose the family whose total MEC score (summed over all offspring) is the smallest.

References

Berger, E., Yorukoglu, D., Peng, J., and Berger, B. (2014). HapTree: A novel Bayesian framework for single individual polyplotyping using NGS data. *PLoS Computational Biology*, **10**(3), e1003502.

Bourke, P. M., Voorrips, R. E., Visser, R. G., and Maliepaard, C. (2015). The double-reduction landscape in tetraploid potato as revealed by a high-density linkage map. *Genetics*, **201**(3), 853–863.

Bourke, P. M., Arens, P., Voorrips, R. E., Esselink, G. D., Koning-Boucoiran, C. F., van't Westende, W. P., Santos Leonardo, T., Wissink, P., Zheng, C., Geest, G., *et al.* (2017). Partial preferential chromosome pairing is genotype dependent in tetraploid rose. *The Plant Journal*, **90**(2), 330–343.

Edgar, R. C. and Flyvbjerg, H. (2015). Error filtering, pair assembly and error correction for next-generation sequencing reads. *Bioinformatics*, **31**(21), 3476–3482.

Karp, A., Nelson, R., Thomas, E., and Bright, S. (1982). Chromosome variation in protoplast-derived potato plants. *TAG Theoretical and Applied Genetics*, **63**(3), 265–272.

Lippert, R., Schwartz, R., Lancia, G., and Istrail, S. (2002). Algorithmic strategies for the single nucleotide polymorphism haplotype assembly problem. *Briefings in Bioinformatics*, **3**(1), 23–31.

Motazedi, E., de Ridder, D., Finkers, R., Baldwin, S., Thomson, S., Monaghan, K., and Maliepaard, C. (2018). TriPoly: haplotype estimation for polyploids using sequencing data of related individuals. *Bioinformatics*, **34**(22), 3864–3872.
