# Supplementary material for: Family-Based Haplotype Estimation and Allele Dosage Correction for Polyploids Using Short Sequence Reads
Source: Front Genet. 2019 Apr 16;10:335. doi: 10.3389/fgene.2019.00335 (PMC6477055; doi:10.3389/fgene.2019.00335)
Supplement: Supplementary file 5 [file Data_Sheet_5.pdf]

Figure S1

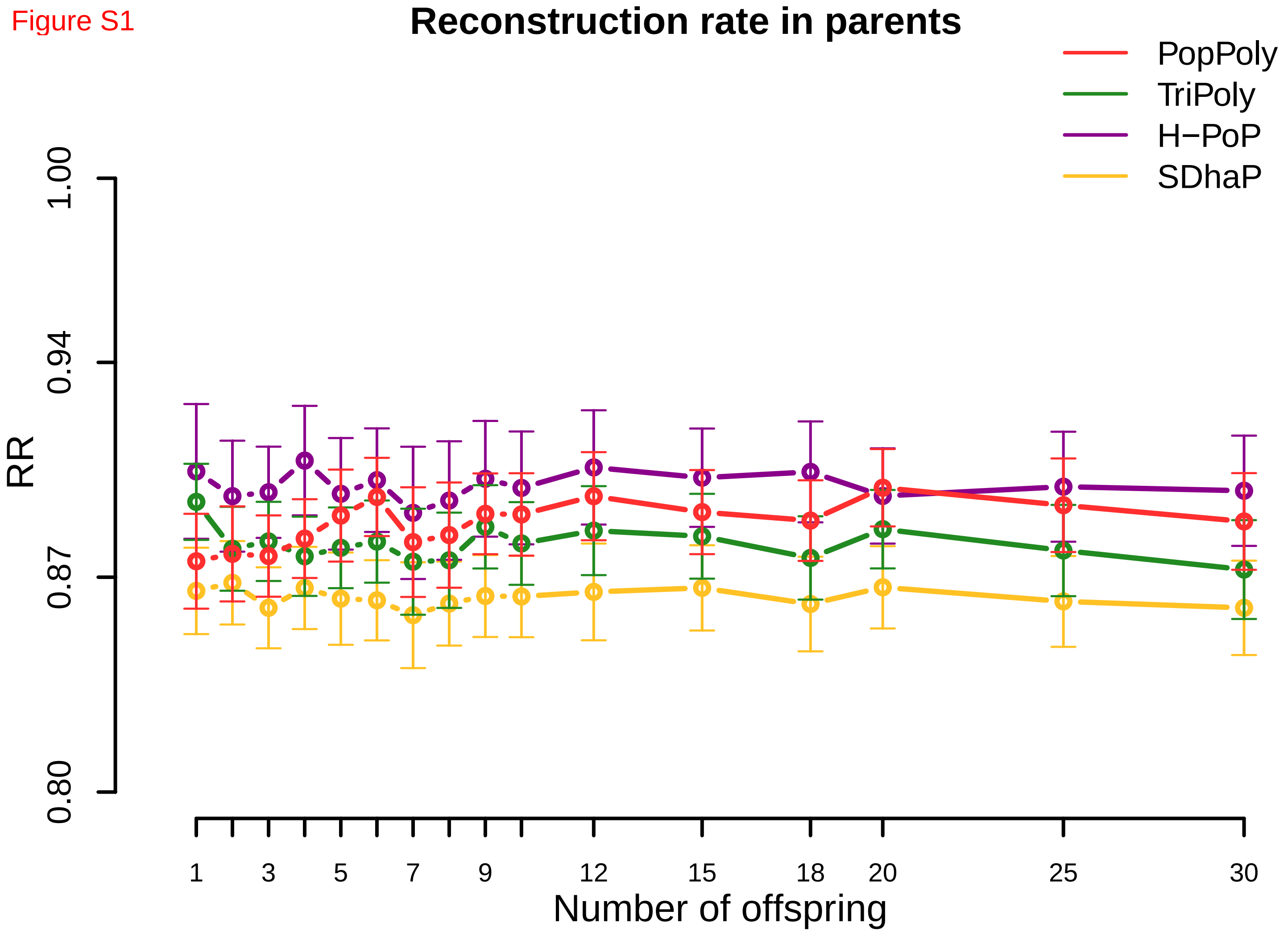
Reconstruction rate in parents
PopPoly
TriPoly
H−PoP
SDhaP
RR
1.00
0.94
0.87
0.80
1
3
5
7
9
12
15
18
20
25
30
Number of offspring

Figure S2

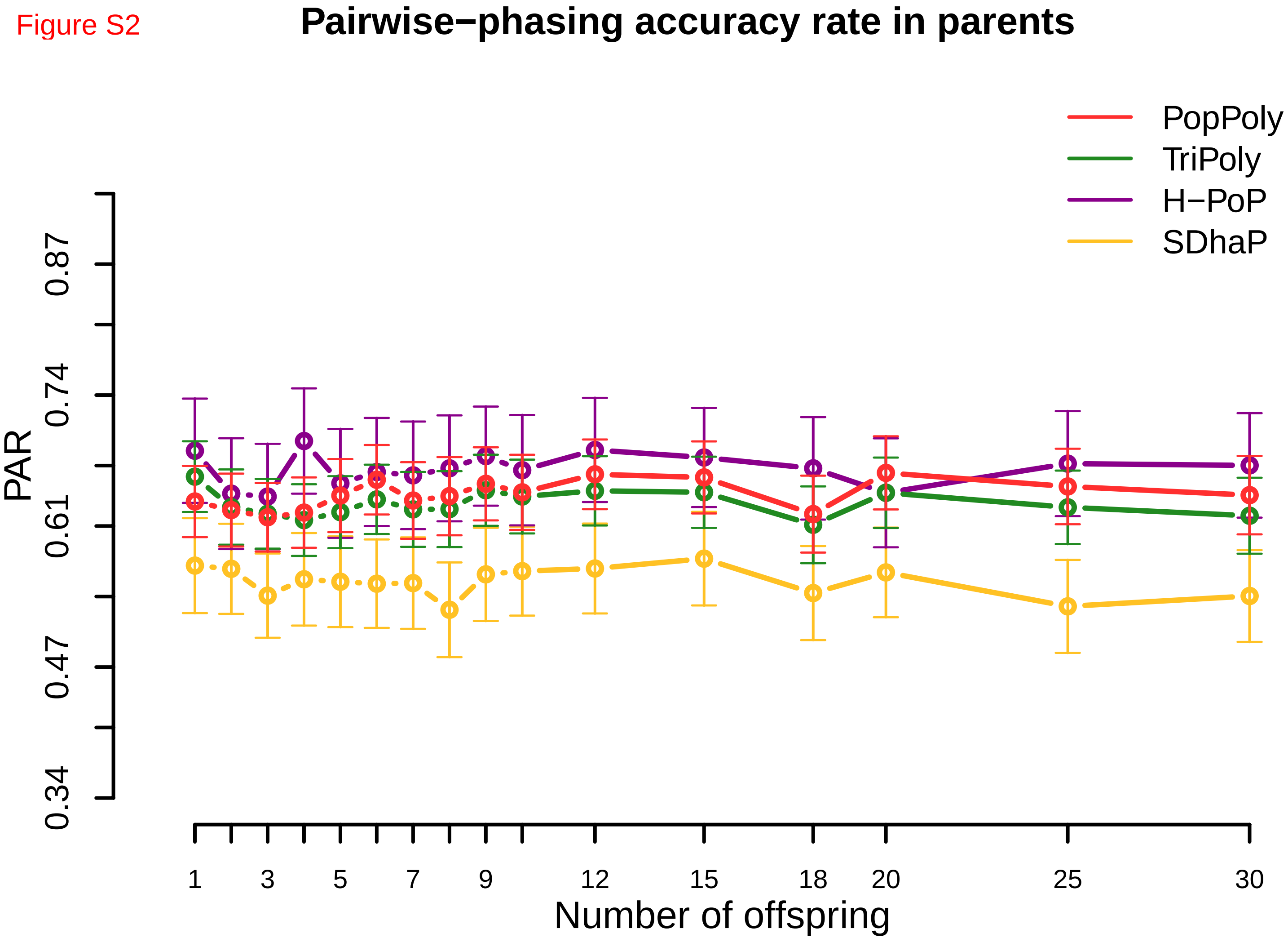
Pairwise−phasing accuracy rate in parents
PopPoly
TriPoly
H−PoP
SDhaP
PAR
0.34
0.47
0.61
0.74
0.87
1
3
5
7
9
12
15
18
20
25
30
Number of offspring

Figure S3

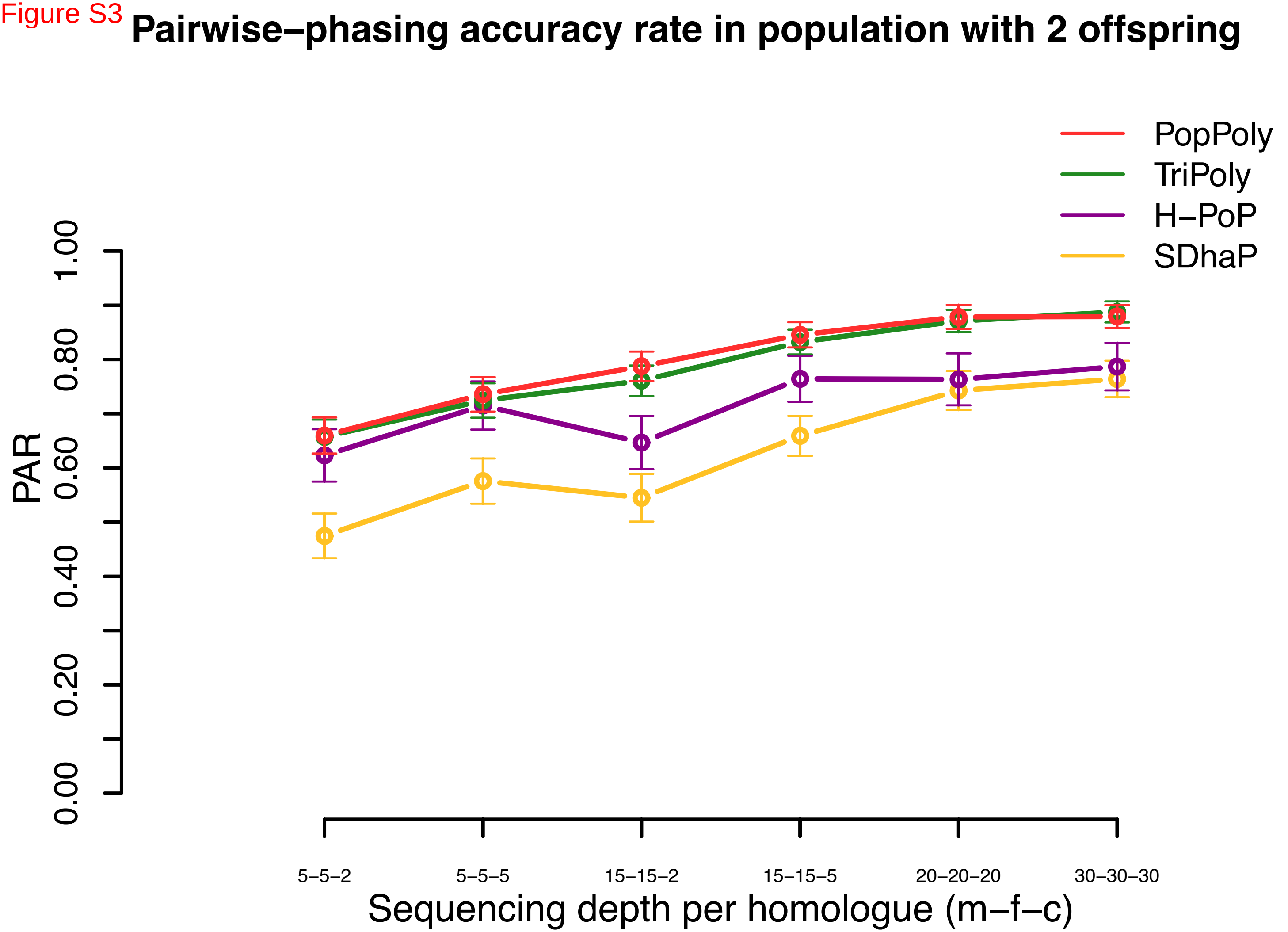
Pairwise–phasing accuracy rate in population with 2 offspring
PopPoly
TriPoly
H–PoP
SDhaP
PAR
0.00
0.20
0.40
0.60
0.80
1.00
5–5–2
5–5–5
15–15–2
15–15–5
20–20–20
30–30–30
Sequencing depth per homologue (m–f–c)

Figure S4

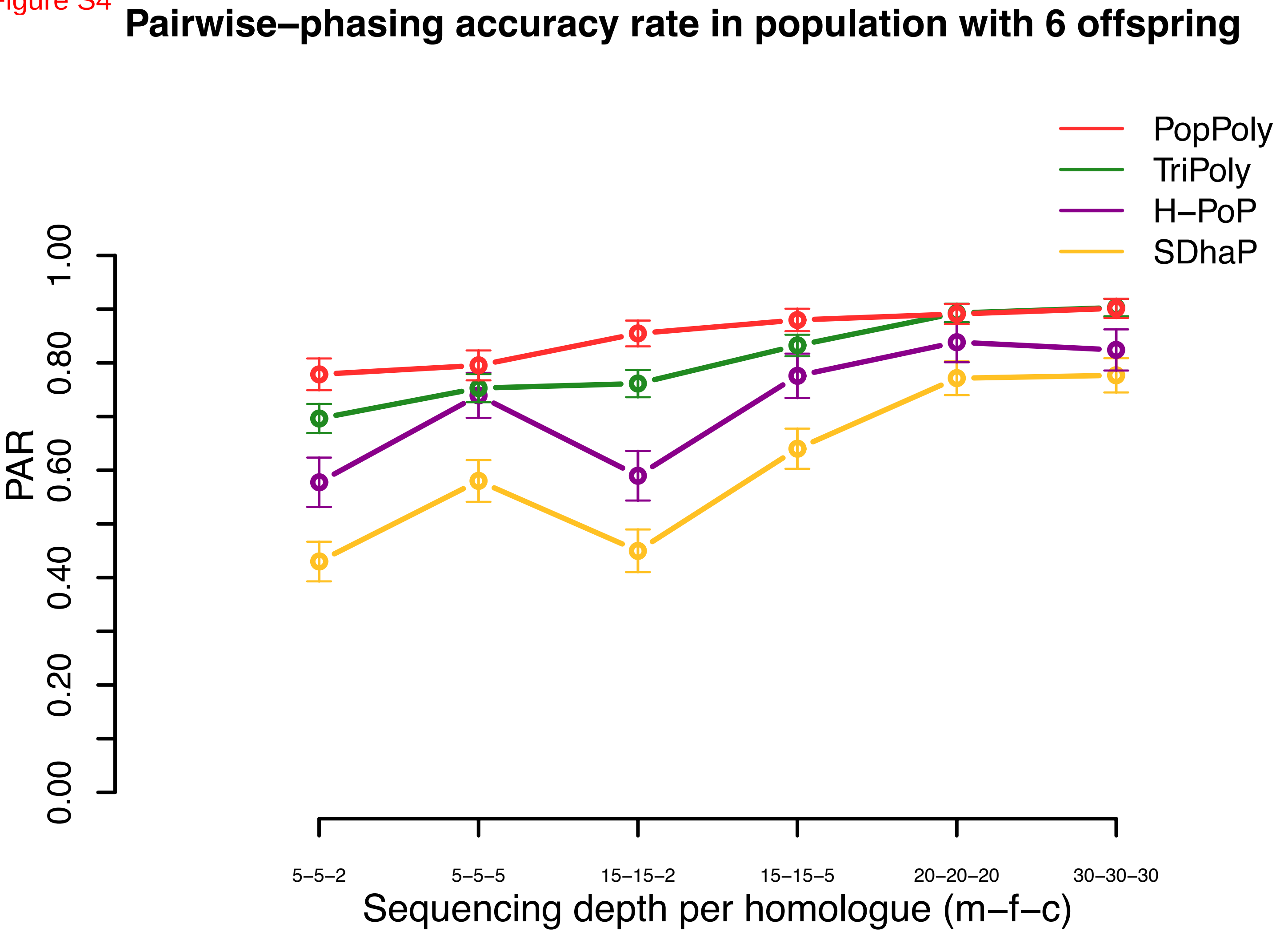
Pairwise−phasing accuracy rate in population with 6 offspring
PopPoly
TriPoly
H−PoP
SDhaP
PAR
0.00
0.20
0.40
0.60
0.80
1.00
5−5−2
5−5−5
15−15−2
15−15−5
20−20−20
30−30−30
Sequencing depth per homologue (m−f−c)

Figure S5

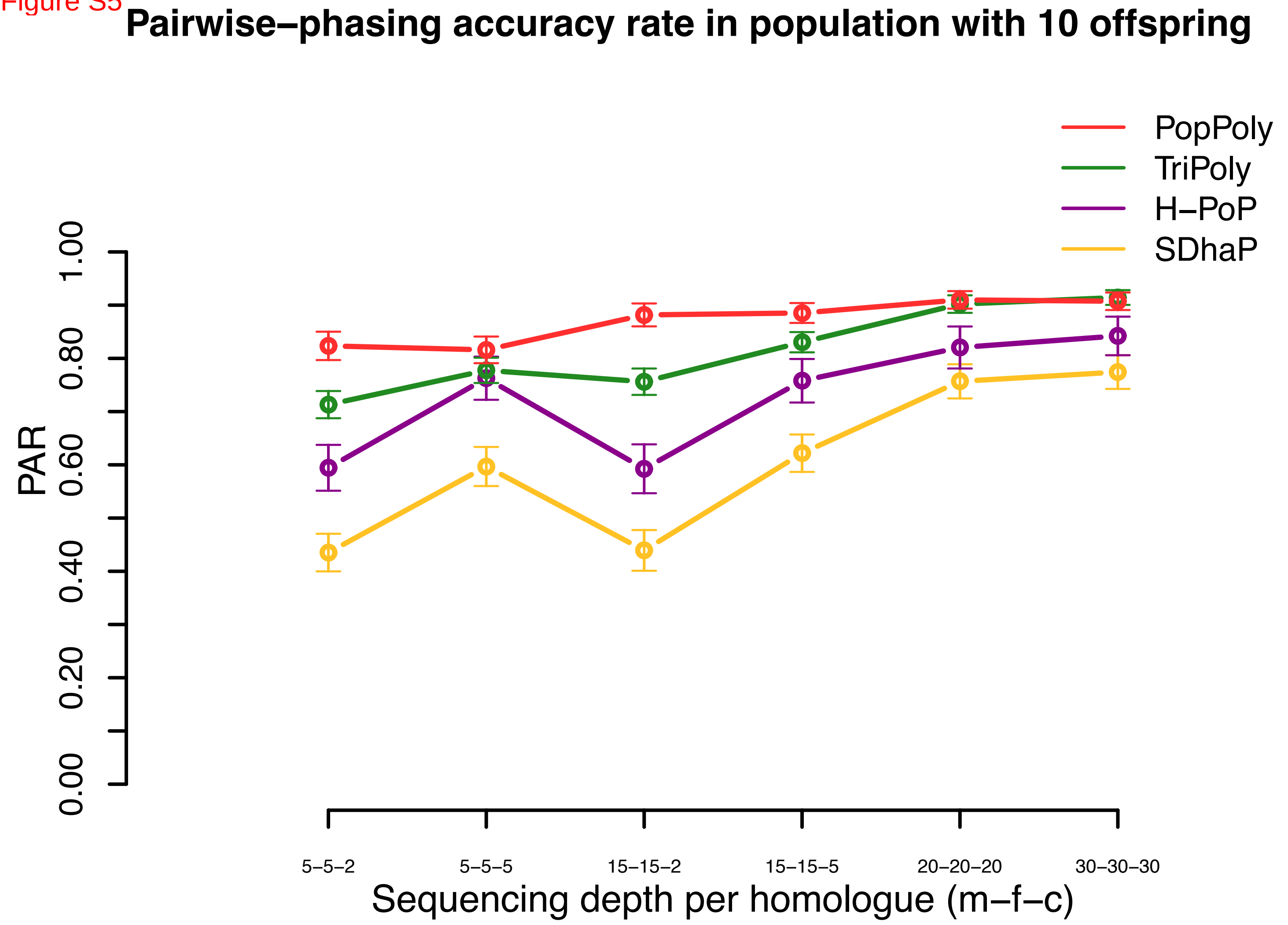
Pairwise–phasing accuracy rate in population with 10 offspring
PopPoly
TriPoly
H–PoP
SDhaP
PAR
0.00
0.20
0.40
0.60
0.80
1.00
5–5–2
5–5–5
15–15–2
15–15–5
20–20–20
30–30–30
Sequencing depth per homologue (m–f–c)

Figure S6

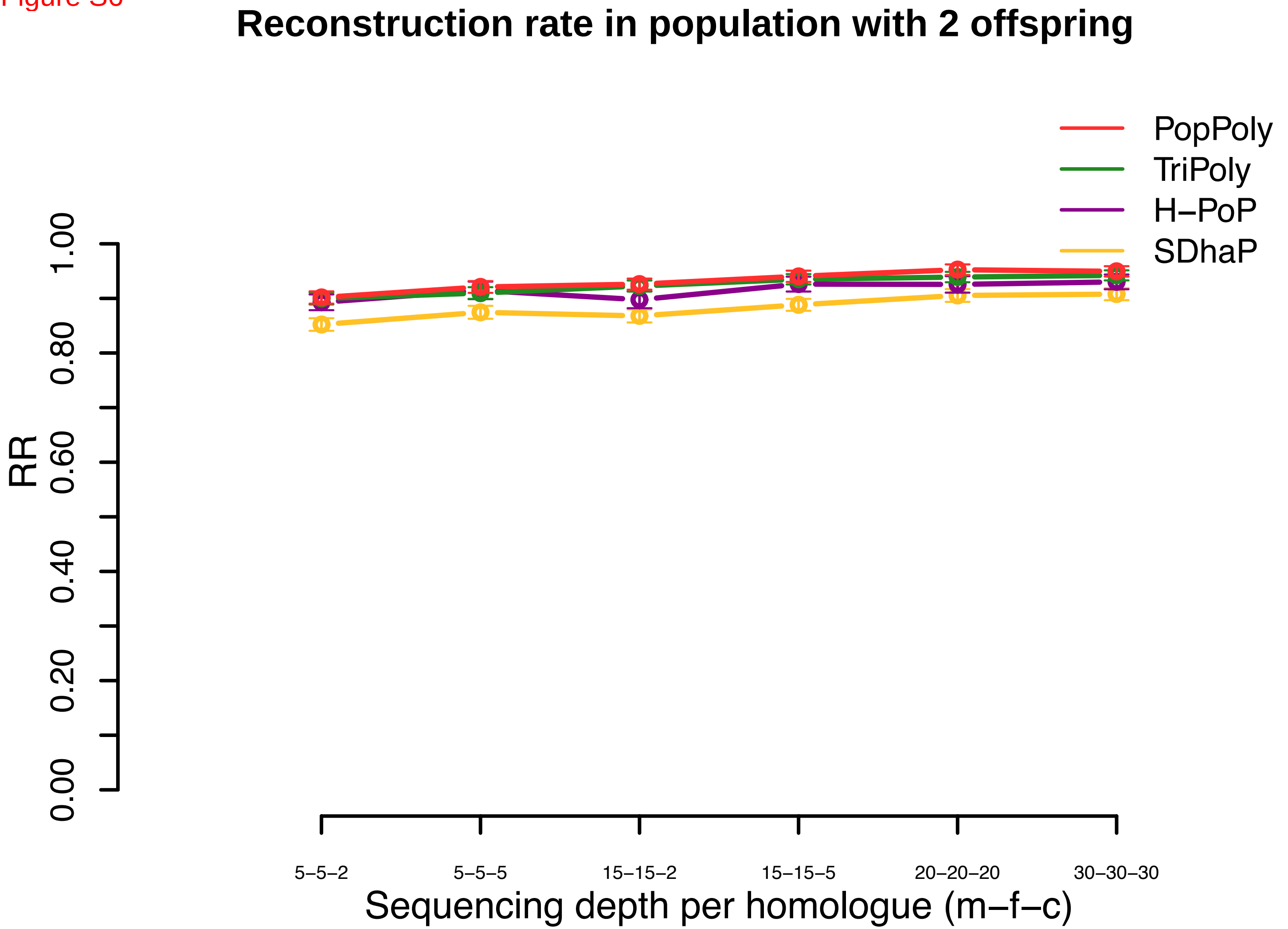

Reconstruction rate in population with 2 offspring
PopPoly
TriPoly
H−PoP
SDhaP
RR
0.00
0.20
0.40
0.60
0.80
1.00
5−5−2
5−5−5
15−15−2
15−15−5
20−20−20
30−30−30
Sequencing depth per homologue (m−f−c)

Figure S7

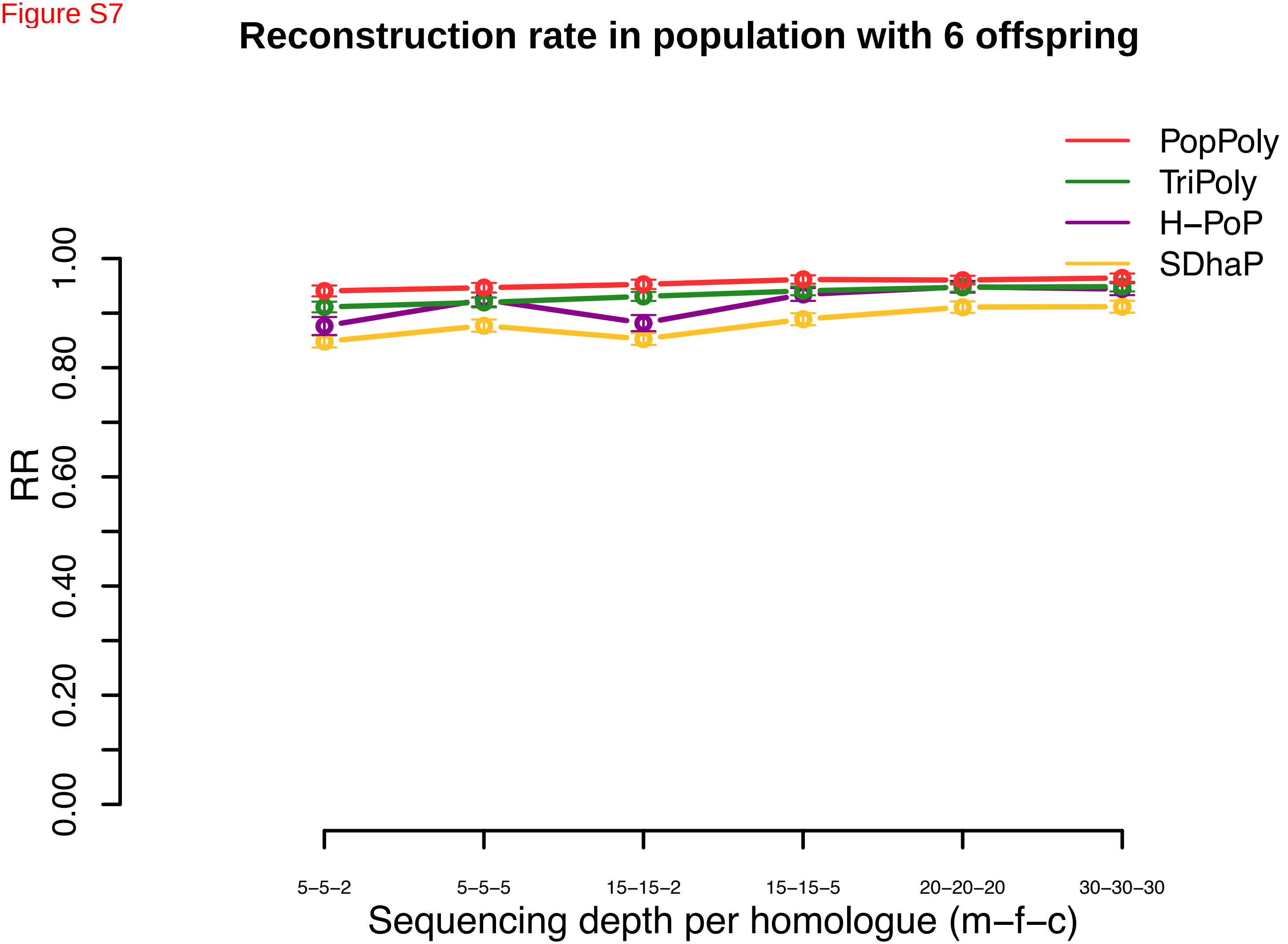
Reconstruction rate in population with 6 offspring
PopPoly
TriPoly
H−PoP
SDhaP
RR
0.00
0.20
0.40
0.60
0.80
1.00
5−5−2
5−5−5
15−15−2
15−15−5
20−20−20
30−30−30
Sequencing depth per homologue (m−f−c)

Figure S8

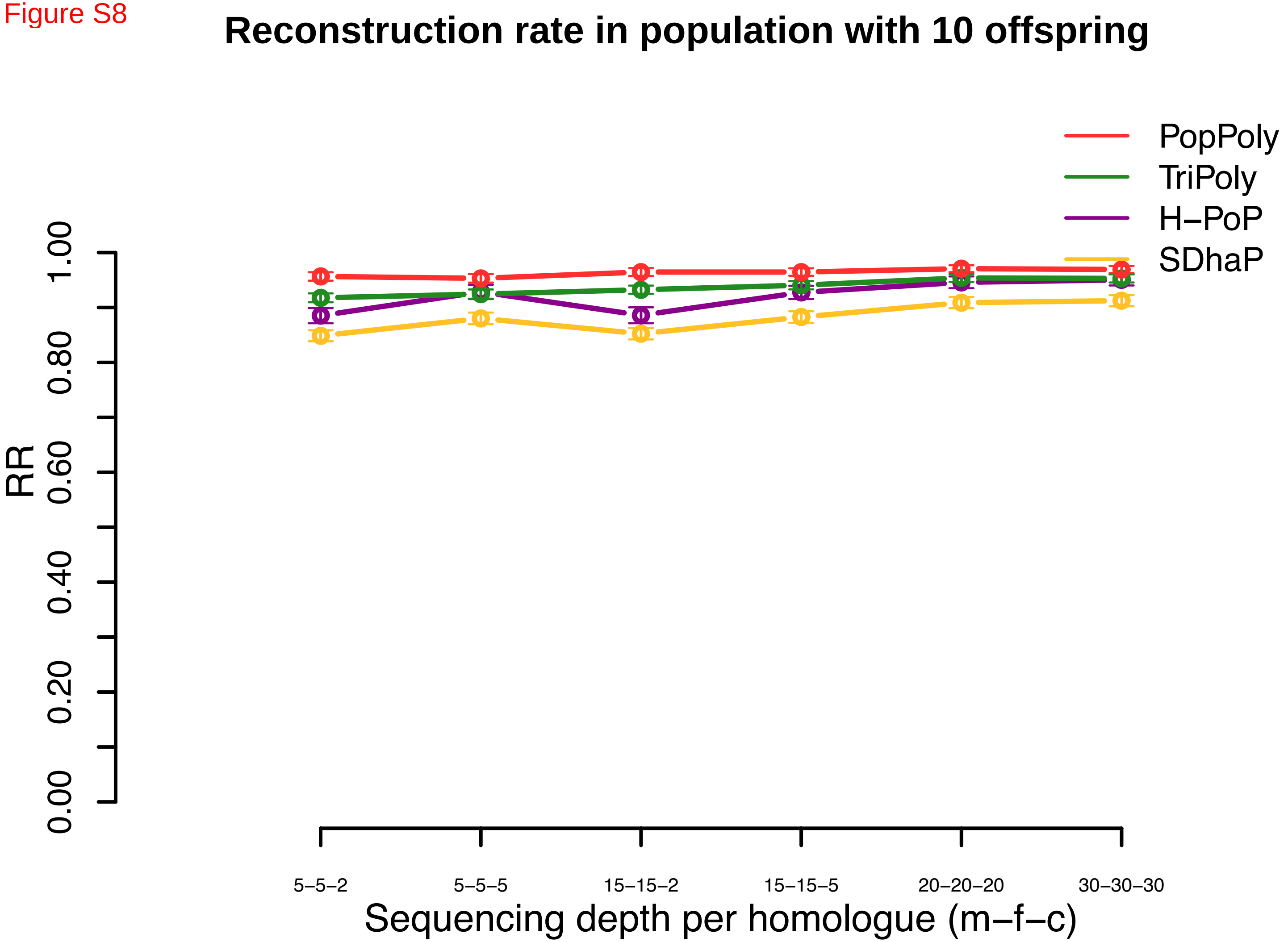

Reconstruction rate in population with 10 offspring
PopPoly
TriPoly
H−PoP
SDhaP
RR
1.00
0.80
0.60
0.40
0.20
0.00
5−5−2
5−5−5
15−15−2
15−15−5
20−20−20
30−30−30
Sequencing depth per homologue (m−f−c)

Figure S9

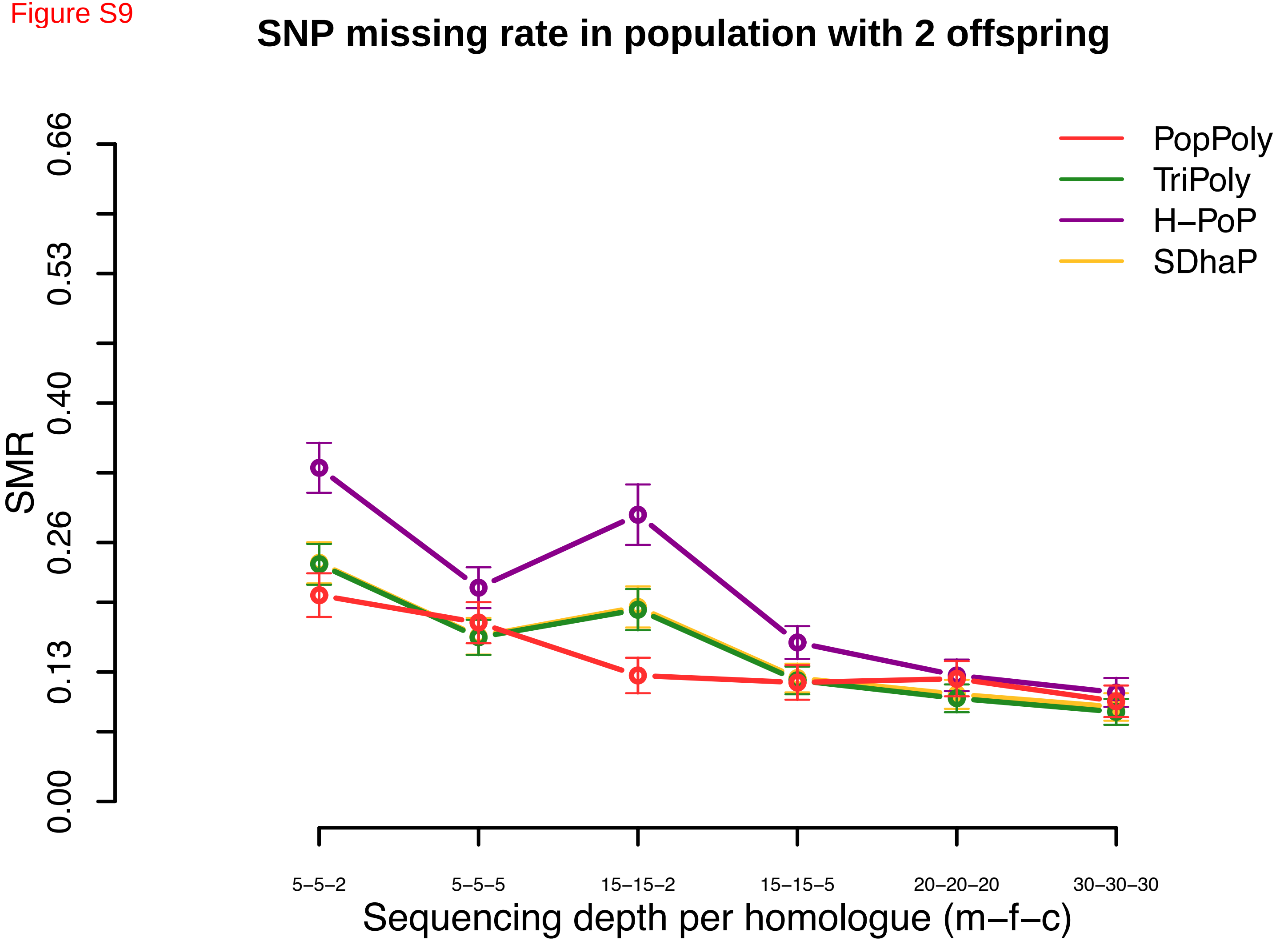
SNP missing rate in population with 2 offspring
SMR
0.00
0.13
0.26
0.40
0.53
0.66
PopPoly
TriPoly
H−PoP
SDhaP
5−5−2
5−5−5
15−15−2
15−15−5
20−20−20
30−30−30
Sequencing depth per homologue (m−f−c)

Figure S10

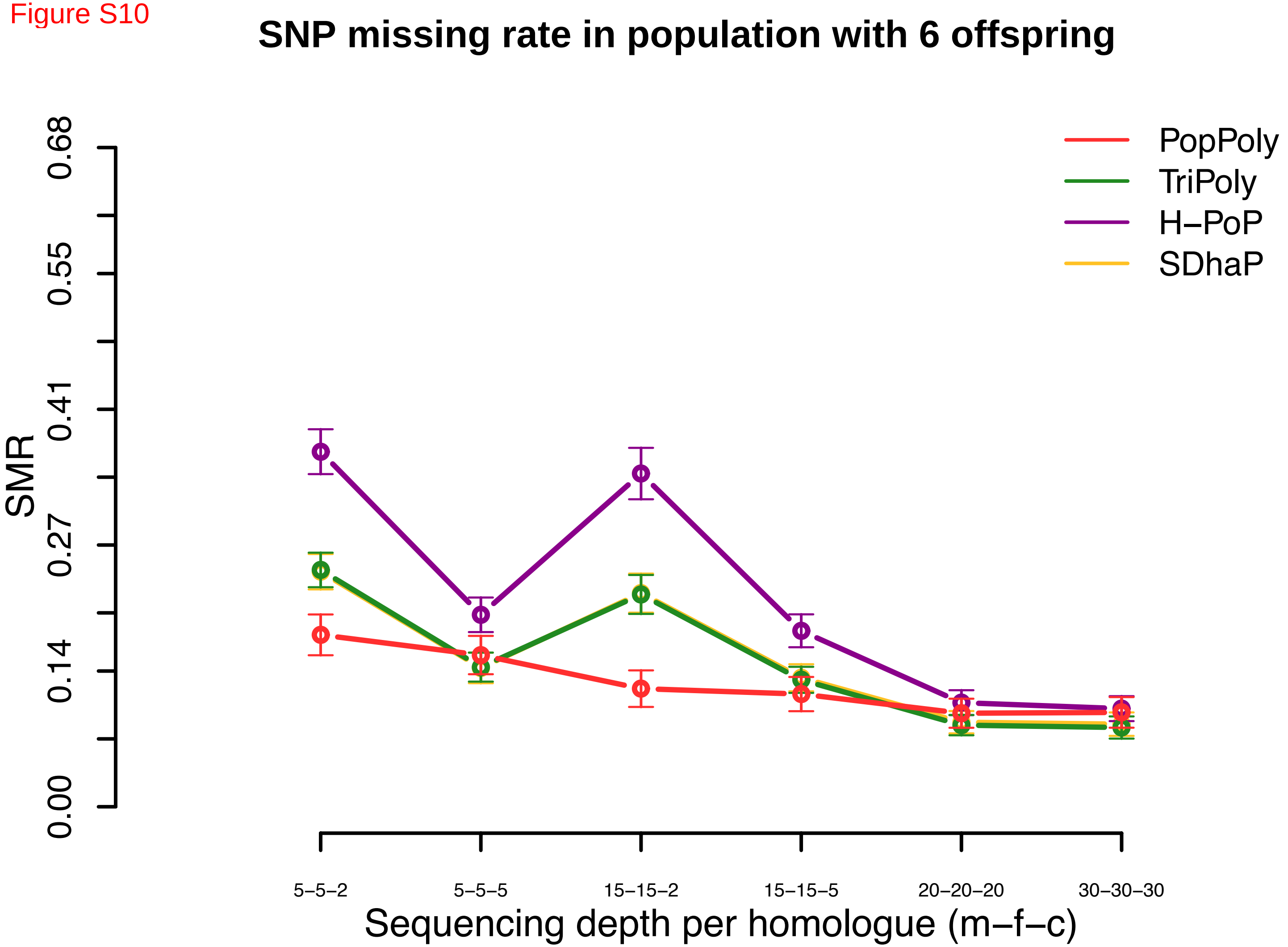
SNP missing rate in population with 6 offspring
SMR
0.68
0.55
0.41
0.27
0.14
0.00
PopPoly
TriPoly
H−PoP
SDhaP
5−5−2
5−5−5
15−15−2
15−15−5
20−20−20
30−30−30
Sequencing depth per homologue (m−f−c)

Figure S11

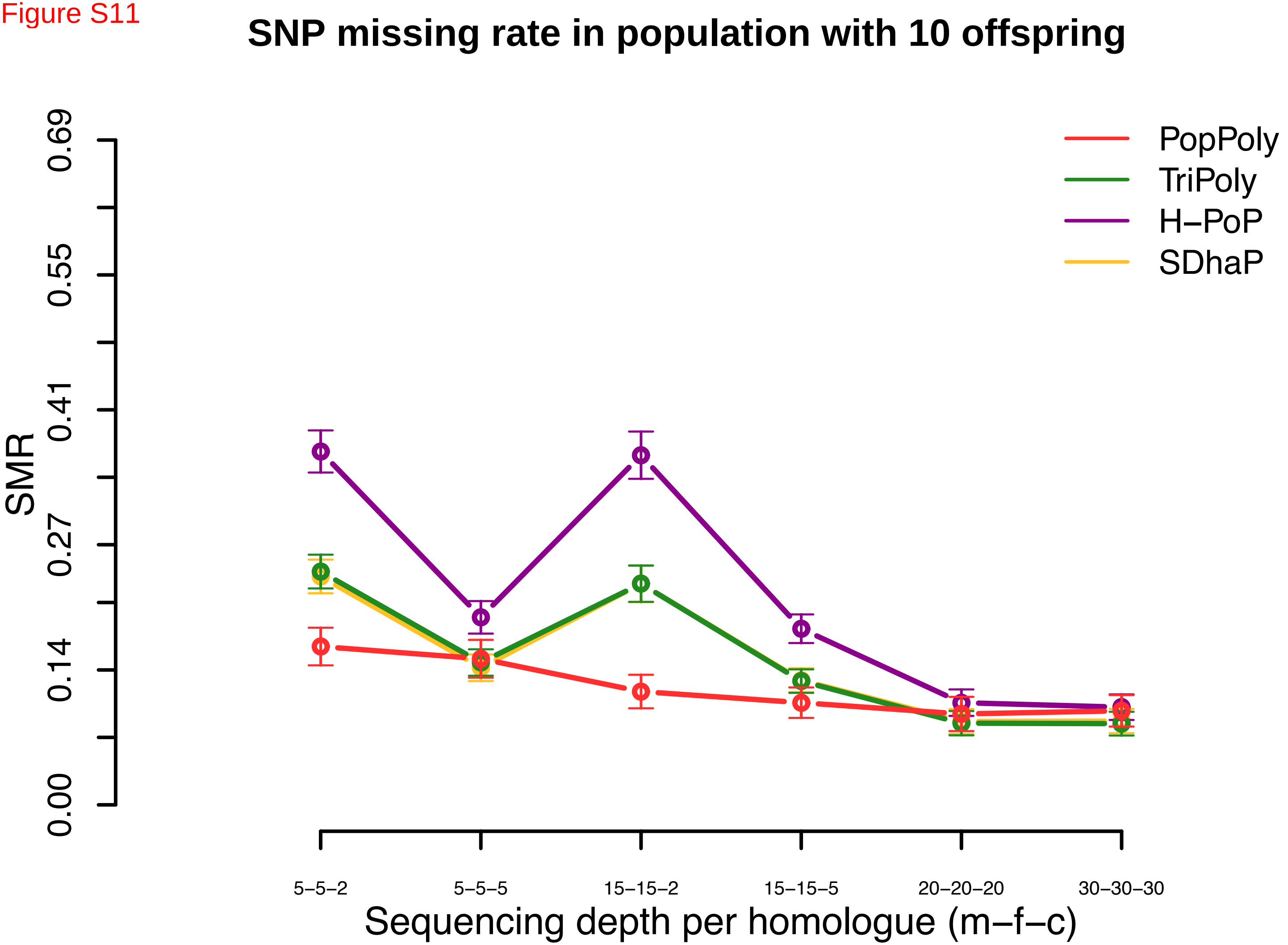
SNP missing rate in population with 10 offspring
PopPoly
TriPoly
H−PoP
SDhaP
SMR
0.00
0.14
0.27
0.41
0.55
0.69
5−5−2
5−5−5
15−15−2
15−15−5
20−20−20
30−30−30
Sequencing depth per homologue (m−f−c)

Figure S12

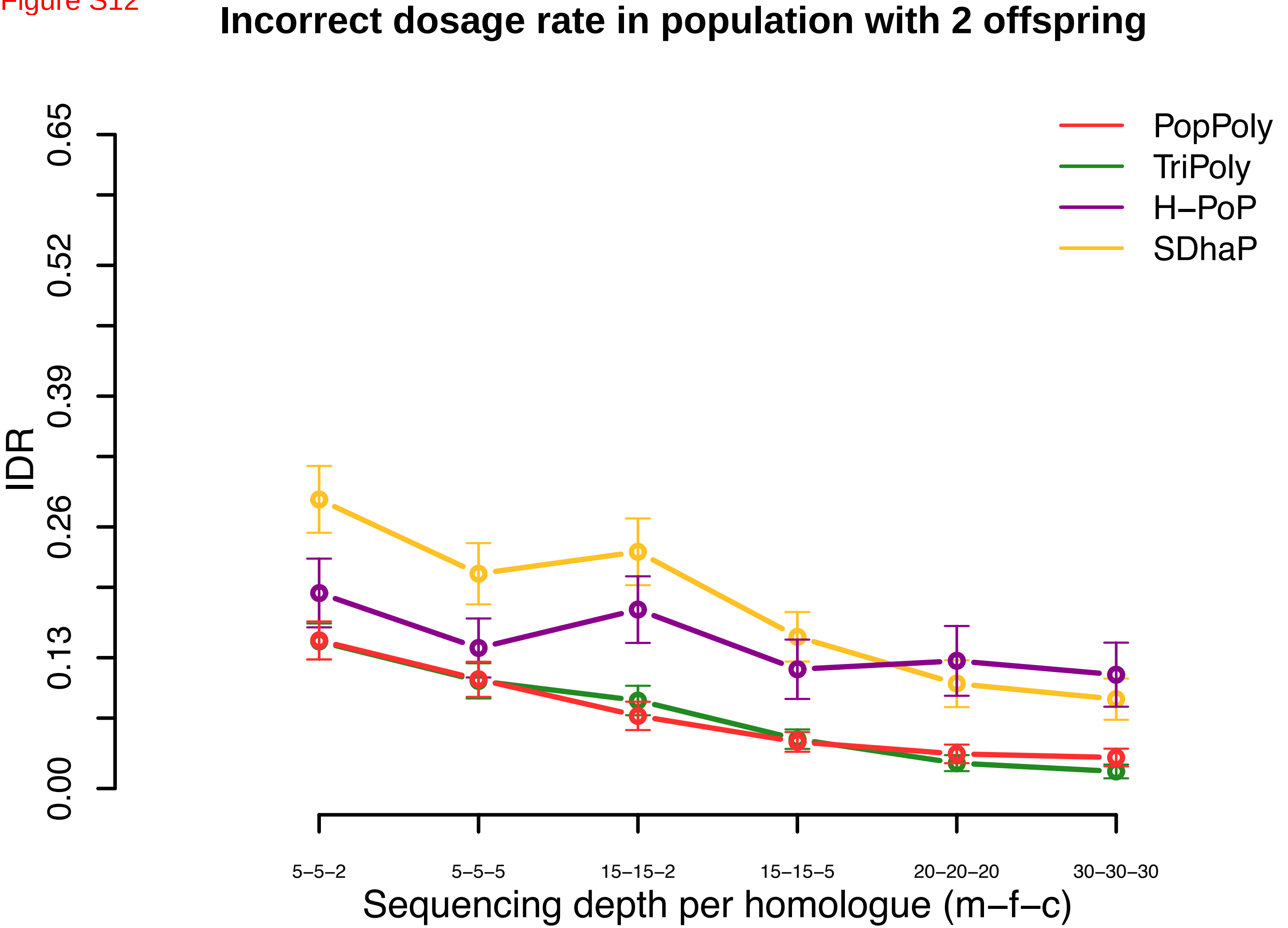
Incorrect dosage rate in population with 2 offspring
IDR
0.00
0.13
0.26
0.39
0.52
0.65
PopPoly
TriPoly
H−PoP
SDhaP
5−5−2
5−5−5
15−15−2
15−15−5
20−20−20
30−30−30
Sequencing depth per homologue (m−f−c)

Figure S13

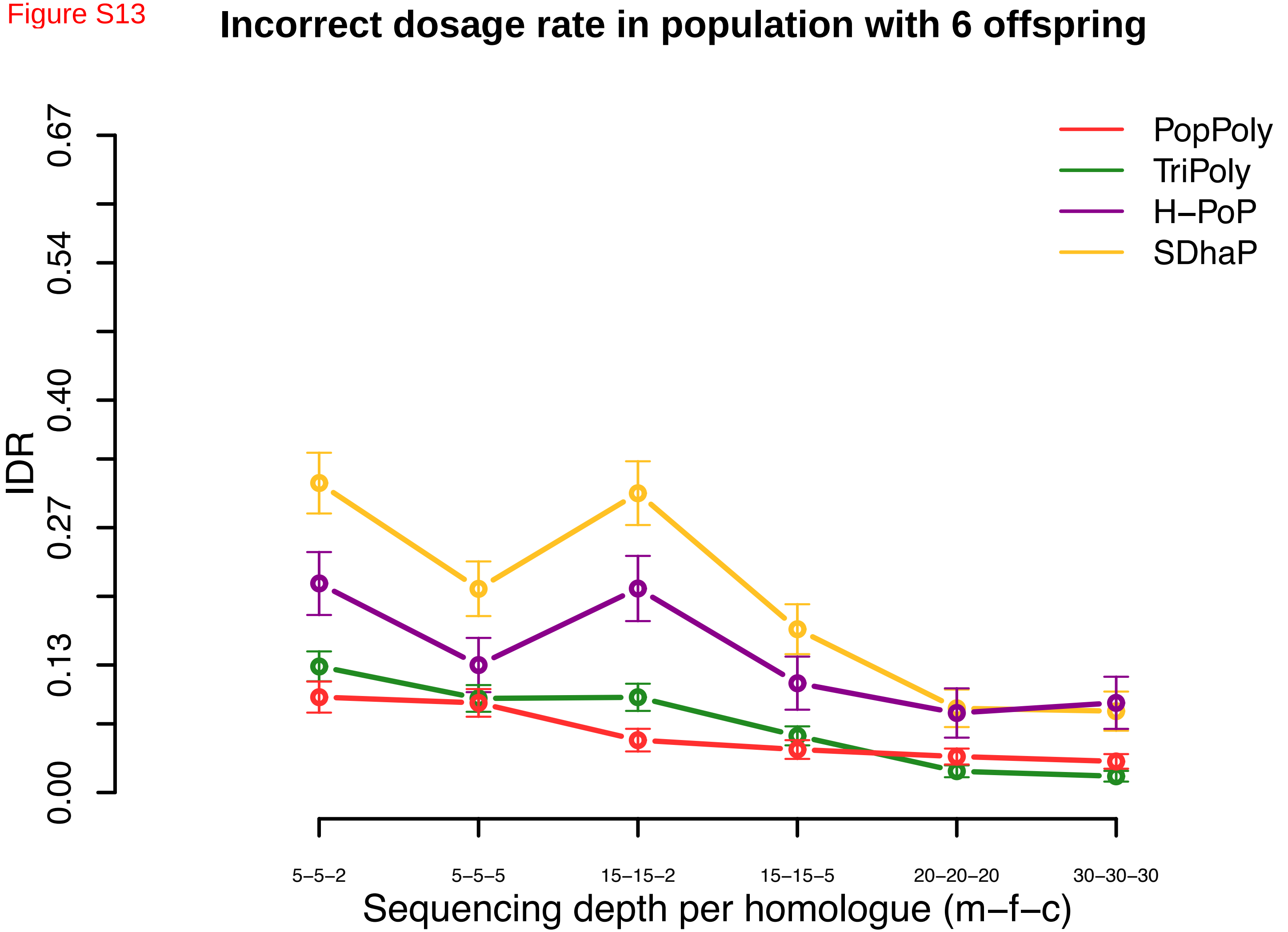

Incorrect dosage rate in population with 6 offspring
IDR
0.00
0.13
0.27
0.40
0.54
0.67
PopPoly
TriPoly
H−PoP
SDhaP
5−5−2
5−5−5
15−15−2
15−15−5
20−20−20
30−30−30
Sequencing depth per homologue (m−f−c)

Figure S14

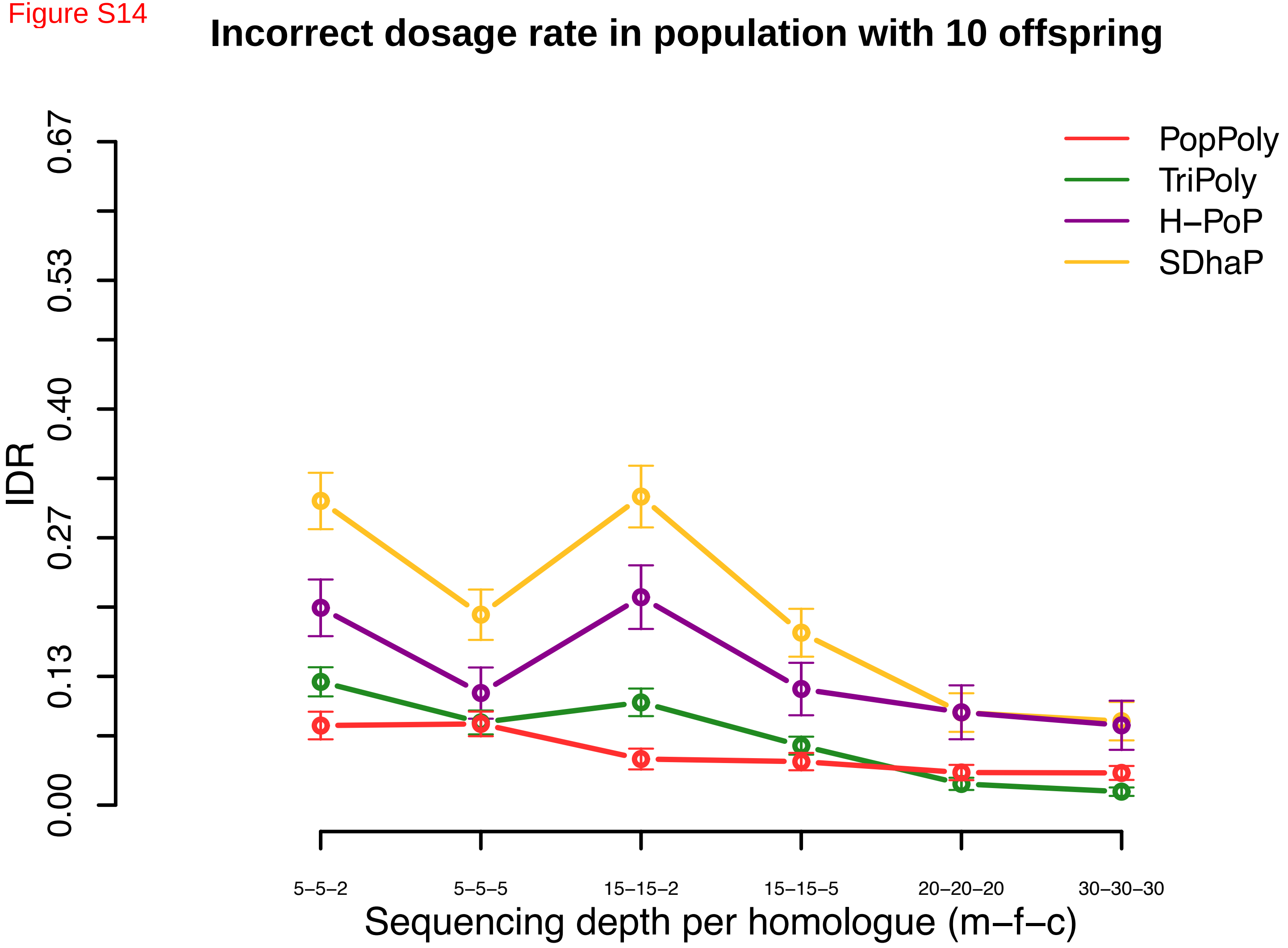
Incorrect dosage rate in population with 10 offspring
IDR
0.00
0.13
0.27
0.40
0.53
0.67
PopPoly
TriPoly
H−PoP
SDhaP
5−5−2
5−5−5
15−15−2
15−15−5
20−20−20
30−30−30
Sequencing depth per homologue (m−f−c)

Figure S15

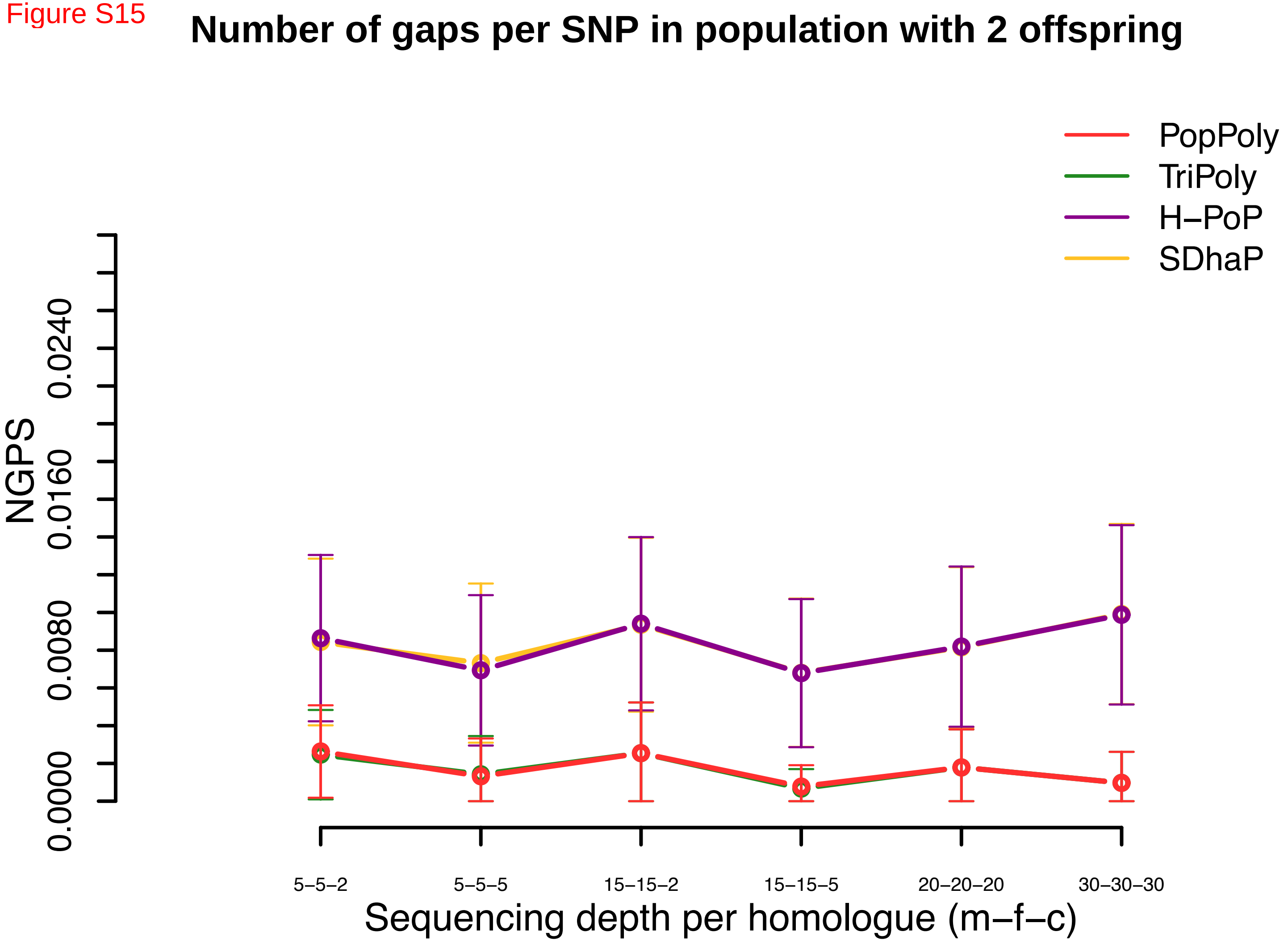
Number of gaps per SNP in population with 2 offspring
PopPoly
TriPoly
H−PoP
SDhaP
NGPS
0.0000
0.0080
0.0160
0.0240
5−5−2
5−5−5
15−15−2
15−15−5
20−20−20
30−30−30
Sequencing depth per homologue (m−f−c)

Figure S16

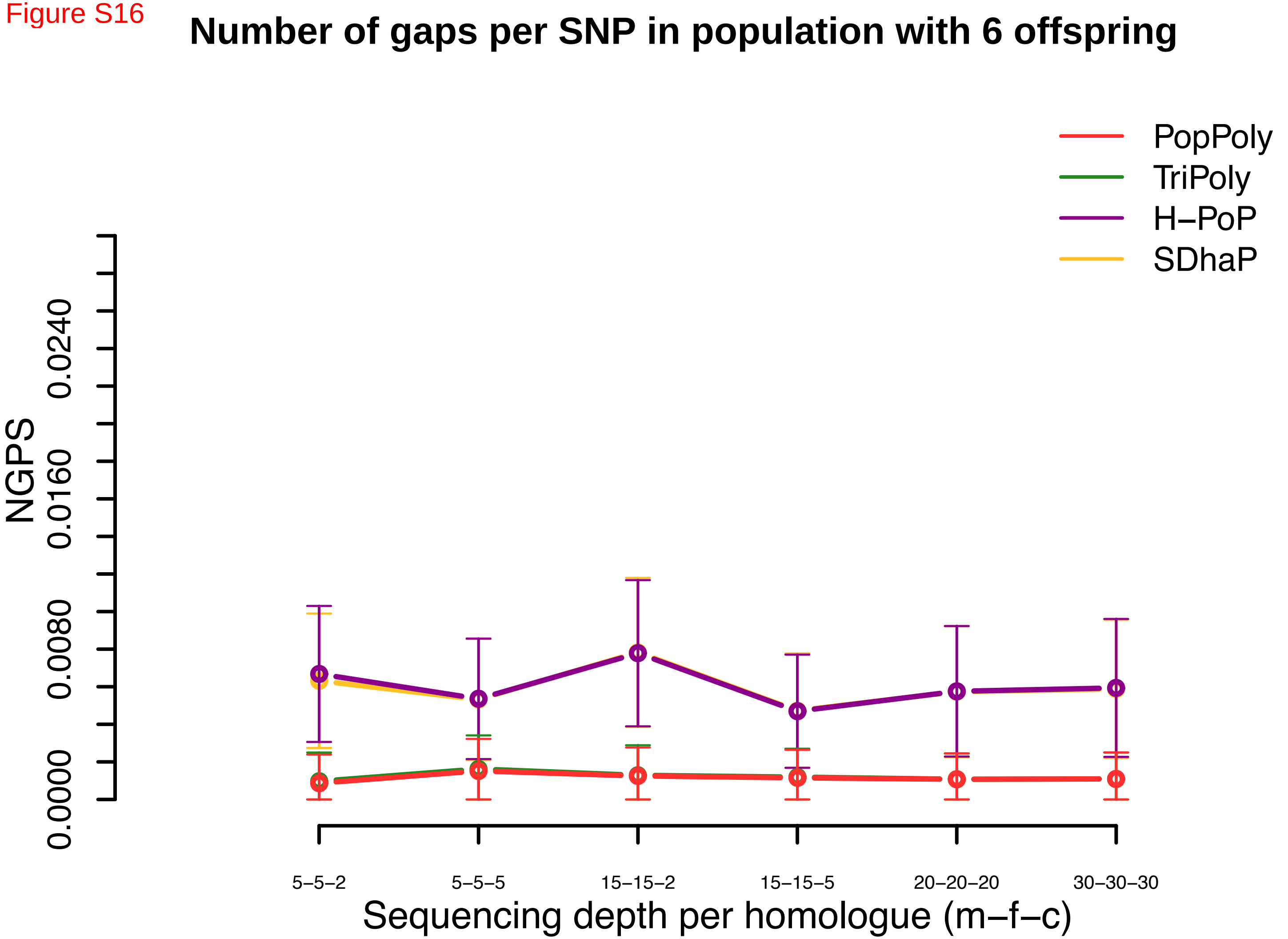

Number of gaps per SNP in population with 6 offspring
PopPoly
TriPoly
H−PoP
SDhaP
NGPS
0.0000
0.0080
0.0160
0.0240
5−5−2
5−5−5
15−15−2
15−15−5
20−20−20
30−30−30
Sequencing depth per homologue (m−f−c)

Figure S17

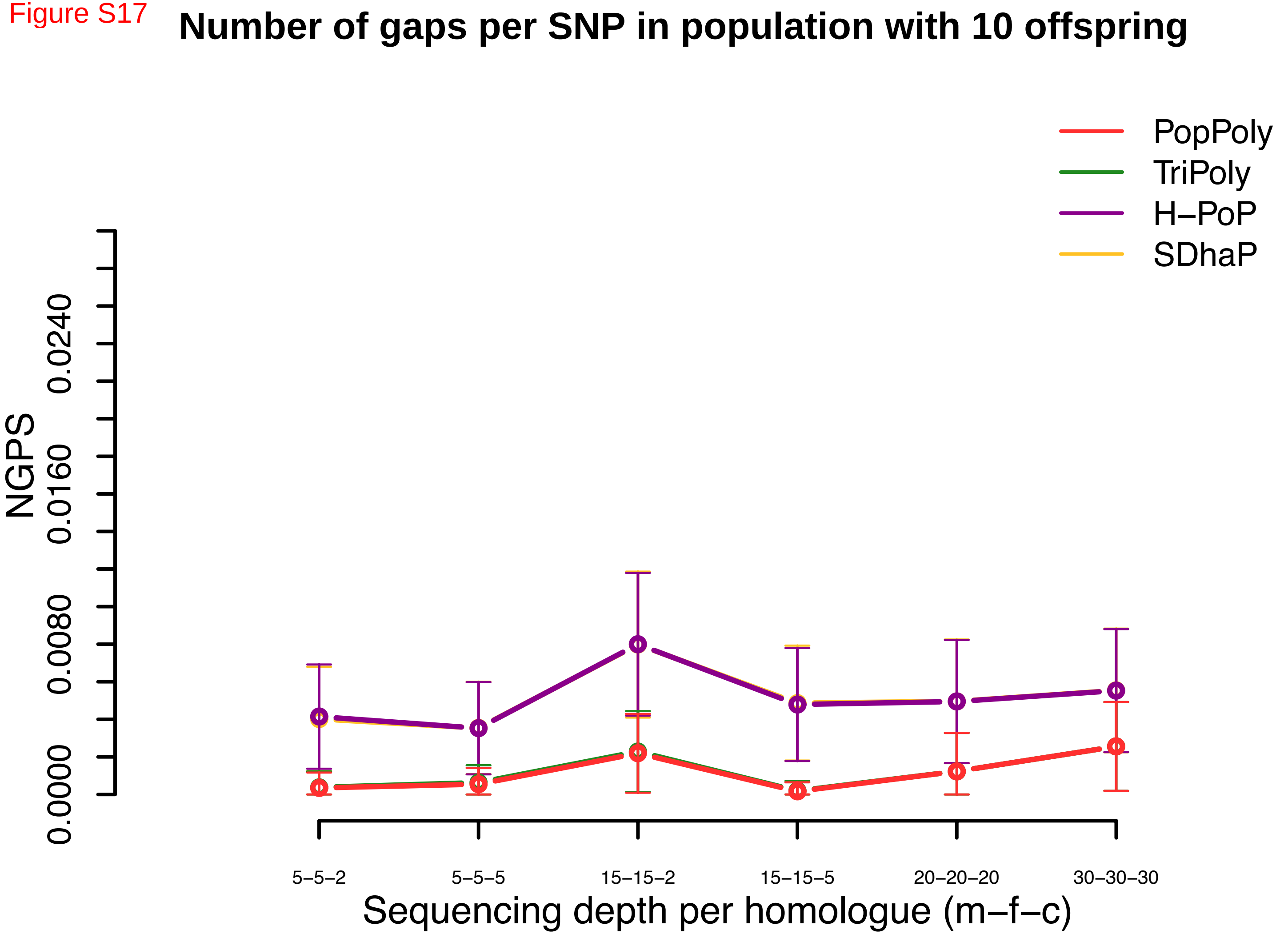

Number of gaps per SNP in population with 10 offspring
PopPoly
TriPoly
H−PoP
SDhaP
NGPS
0.0000
0.0080
0.0160
0.0240
5−5−2
5−5−5
15−15−2
15−15−5
20−20−20
30−30−30
Sequencing depth per homologue (m−f−c)

Figure S18

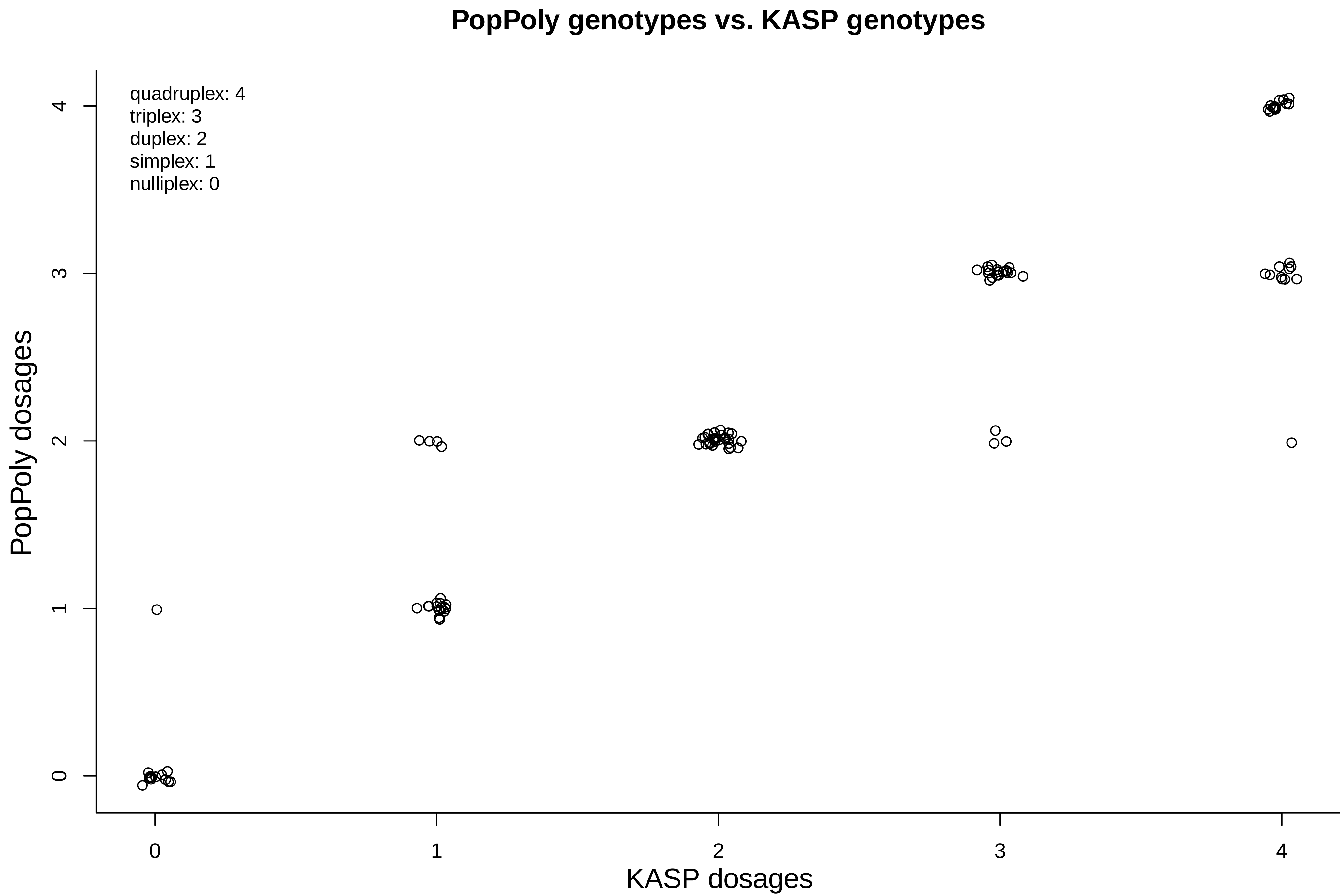
PopPoly genotypes vs. KASP genotypes
quadruplex: 4
triplex: 3
duplex: 2
simplex: 1
nulliplex: 0
PopPoly dosages
KASP dosages
0
1
2
3
4
